# Investigating the Absorption Properties of Pure and Nitrogen-Doped Carbon Clusters as Models for the Core of Carbon Nanodots

**DOI:** 10.1021/acs.jpca.5c07923

**Published:** 2026-02-04

**Authors:** Francesca D’Ambrosio, Alice Frustaci, Alessandro Azzali, Enrico Bodo

**Affiliations:** Chemistry Department, 9311 University of Rome “La Sapienza”,P. Aldo Moro 5, Rome 00185, Italy

## Abstract

The study of amorphous carbon structures of different sizes and extensions is relevant to many research areas, including electrode processes (e.g., intercalation), astrochemistry, catalysis, and sensors. While the structure of amorphous carbon structures has been investigated thoroughly in the past, a systematic analysis of their properties upon doping with functional groups is far less extensive. This aspect is particularly important for carbon nanodots (CNDs), a photoluminescent species of carbon-based nanoparticles whose optical properties arise from the interplay between core electronic structure, surface states, heteroatom doping, and molecular fluorophores. Despite extensive experimental work, an atomistic rationalization of their optical properties is still not available. In this study, we adopt a bottom-up computational approach using amorphous pure carbon clusters (C_10_–C_60_) and nitrogen-substituted ones (C_9_N–C_59_N) as models for the unsaturated and partially doped domains of CND cores. Structural isomers were generated along with computed UV/vis spectra to rationalize the property changes upon nitrogen substitution.

## Graphical abstract

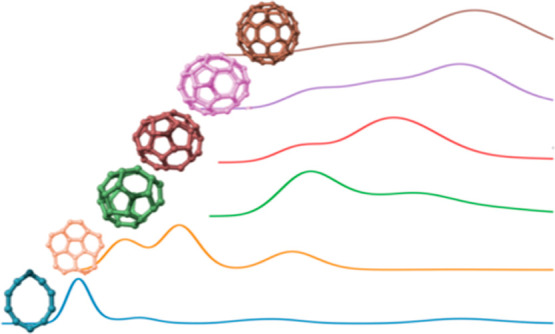

## 1 Introduction

Carbon nanoparticles show a wide spectrum of forms whose properties are governed by atomic hybridization, size, and degree of structural order. Among these, amorphous carbon nanoparticles are characterized by a lack of long-range order, by a mixture of sp^2^/sp^3^ domains, by a rich surface chemistry, and by the presence of structural defects. Such a disordered form of carbon has been increasingly recognized as a subject of renewed interest in recent years.^1,2^

In astrochemistry, amorphous carbon nanoparticles are considered one of the main constituents of cosmic dust and interstellar solid matter. Their imprints on the observed radiation include absorption and scattering of starlight, while their catalytic effect is believed to provide an efficient route for (relatively) complex molecular synthesis. They also influence the thermal equilibrium in interstellar clouds.^3,4^

Amorphous carbon species are among the materials used for developing electrodes and other energy-related materials. The high surface area, tunable conductivity, and chemical stability make these materials active components in supercapacitors, batteries, fuel cells, and sensors.^5,6^

Finally, carbon nanodots (CNDs), sub-nanometric carbon nanoparticles with luminescent properties, typically derived from partially carbonized amorphous precursors, possess peculiar photoluminescent properties induced by their (still not entirely known) internal and superficial structure and by a complex interplay of defect states and surface functional groups. These nanoparticles belong to the more general class of carbon dots, first reported by Xu and co-workers in 2004,^7,8^ which are carbon-based nanoparticles characterized by intense photoluminescence with applications in various fields such as biomedicine,^9–12^ catalysis,^13,14^ and sensor applications.^15–21^

The present study focuses primarily on CNDs, which are quasi-spherical nanoparticles and are composed of a partially amorphous carbonaceous core coexisting with sp^2^-ordered domains and surface functionalization.^22^ A viable model of their atomic-level structure and how it correlates with their properties is still not available.^23^ This lack of knowledge arises from the inherent complexity involved in modeling such systems. A realistic model would have to be large enough to include both the carbonaceous core and the rich functionalized outer layer. It should describe the coalescence of different structural motifs (coexistence of sp^2^ and sp^3^ domains) over a nanometric scale and the surface-rich surface functionalization. In addition, it should also reflect the composition determined by the specific synthetic method, which can vary significantly across experimental techniques. Such a model, especially at the quantum level, is beyond any computational technique available to us.^24^

Initial attempts to model CNDs mostly relied on small aromatic molecules and polycyclic aromatic hydrocarbons (PAHs) or focused on representing the amorphous carbon core through random networks or polymer-like models.^25–27^ However, these models only partially capture the intrinsic complexity of the disordered carbon framework typical of CNDs.^28^

To address this issue, we propose a complementary strategy that involves using amorphous carbon clusters (CCs) as model systems for the amorphous and unsaturated portions of the nanodot’s core. Amorphous CCs have been known as fullerene precursors under high-temperature conditions, undergoing structural rearrangements toward cage-like geometries.^29^ Building on these studies, we hypothesize that progressively larger CCs could serve as effective structural motifs for describing the unsaturated regions of CNDs. As these clusters grow, they tend to adopt more compact, cage-like structures that could assemble within the nanodot core during the energetic phases of its synthesis.

Ultimately, any theoretical model for CNDs should be able to provide optical properties, which are of primary importance. The origin of these properties, especially their excitation-dependent emission, remains unclear, though some aspects have been clarified over the years. CND absorption spectra are generally dominated by a strong π–π* transition in the ultraviolet (230–340 nm) range, which arises from conjugated sp^2^ carbon domains. Less intense *n*–π* bands or visible shoulders (400–450 nm) are associated with surface carbonyl, amide, or nitrogen-containing moieties. The coexistence of these features gives rise to characteristic long absorption tails that extend deep into the visible region. Their photoluminescence (PL) exhibits broad emission bands, large Stokes shifts, and frequently, excitation-dependent behavior. These features indicate the presence of multiple emissive centers rather than a single quantum-confined electronic transition. Several additional factors influence the optical properties of CNDs: their surface chemistry and the presence of molecular fluorophores embedded in the structure. The relative contributions of these factors depend on the synthesis conditions, degree of carbonization, and local environment.^28,30–35^

To rationalize this intricate set of interrelated factors, we adopted a deliberately simplified chemical model designed to disentangle the underlying cause–effect relationships, thus offering a qualitative framework instead of a full model of CNDs. This work focuses particularly on the role of nitrogen doping and on the influence of a simple localized structural modification on optical transitions. Nitrogen doping is known to enhance PL quantum yields and modulate emission color. However, according to systematic studies,^33,36,37^ the effect of nitrogen is not solely due to surface passivation, but rather, it is due to changes in the electronic structure of the carbonaceous core. At moderate doping levels, nitrogen atoms substitute for carbon within sp^2^ domains. These nitrogen atoms act as electron-donating or electron-trapping sites that stabilize surface excitons and lead to efficient blue emission. Sciortino et al.^36^ have found evidence to support such transformations, which directly impact absorption and emission by introducing new low-energy transitions around 410 nm and enabling dual blue/green photoluminescence.

From a theoretical perspective, TD-DFT calculations^37^ have demonstrated that graphitic nitrogen, when embedded within the π-conjugated domain, significantly red-shifts absorption by narrowing the HOMO–LUMO gap and forming new electronic states within the optical gap. In contrast, pyridinic, pyrrolic nitrogen atoms, and amino groups, which are typically located on the edges, exert minor or opposite effects. This distinction explains why some nitrogen-doped CNDs exhibit visible absorption and green-to-red emission while others remain strongly blue-emitting.

Parallel experimental efforts^30,32,38^ have demonstrated that molecular fluorophores and surface states significantly contribute to the overall optical response. During the carbonization of organic precursors, small aromatic fluorophores may form. These molecular species account for the narrow, excitation-independent fluorescence components that are often superimposed on the broader CND emission.^30^ Additional studies^35,39^ have shown that optical behavior is dominated by hybrid sp^2^/sp^3^ architectures and local charge-transfer processes.

In our previous work,^40^ we investigated the most stable isomers of singly doped, neutral, unsaturated CCs with various substituents. In the present study, we extend this approach by focusing on graphitic nitrogen-doped clusters and presenting a comparison of the absorption properties of neat and doped clusters, thus elucidating the influence of nitrogen substitution.

It should be noted that the structures presented here are not intended as full models of CNDs, but rather as minimal, structural motifs designed to probe the electronic and optical effects of unsaturated carbon domains and nitrogen substitution. By intentionally focusing on unsaturated and weakly functionalized CCs, we aim to isolate the properties associated with local motifs that are expected to coexist within the amorphous core of CNDs. This deliberately simplified approach allows us to disentangle the role of nitrogen substitution on the electronic levels and optical transitions of carbon domains, without the confounding influence of surface passivation, molecular fluorophores, solvent effects, or structural disorder. In this way we, gain insights that would otherwise be difficult to obtain from more elaborate, complex models. The relevance of our results lies in identifying trends such as size-dependent redshifts, charge-transfer character, and the emergence of low-energy optically active states upon nitrogen incorporation that are expected to persist once these motifs are incorporated into more realistic CND architectures.

## 2 Methods

The computational protocol chosen for this work was based mostly on the global optimizer algorithm (GOAT)^41^ implemented in the ORCA software (version 6.0),^42,43^ which provided an efficient choice, allowing an extensive isomer and conformer search starting from a given initial structure. To keep computational cost under control, the exploration of the conformational space was carried out using the semiempirical GFN2-xTB method developed by Grimme’s group,^44^ which offers an excellent compromise between accuracy and computational cost.

The initial model structures, containing between 10 and 60 total atoms of carbon, were generated using an in-house code that increases geometric variability by distributing a defined number of carbon atoms within a domain, producing a set of geometries that purposely include monocyclic, polycyclic, and cage-like structures. Each configuration, before being subject to the GOAT search, was preoptimized using the GFN2-xTB method within the XTB software package.^44,45^ The same procedure was applied to nitrogen-doped systems, where one carbon atom of the initial structure is substituted with a nitrogen atom. The initial structures were then fed into the GOAT search, using the variant GOAT-EXPLORE^41^ that allows bond topology changes. The GOAT final ensemble of optimized structures spans an energy window of approximately 60 kcal mol^–1^, corresponding to the difference between the lowest- and highest-energy isomers identified (see Section 3.2.1, for a discussion of these ensembles). We note here for clarity that the GOAT algorithm does not model the kinetics of the carbonization process itself but provides only a systematic exploration of low-energy structures compatible with a given atomic composition.

From these resulting ensembles of xTB structures, 3 to 6 structurally distinct, low-energy isomers were hand-picked and further optimized at the ωB97X-D3/def2-TZVP level of theory.

The resulting DFT structures for a selection of cluster sizes (10, 20, 30, 40, 50, and 60 atoms) were used for the computation of absorption spectra, performed at the ωB97X/def2-TZVP level using TD-DFT.^46–51^ All TD-DFT calculations were performed using the Tamm–Dancoff approximation, as per Orca’s default.^52^ Coulomb and exchange terms were treated using the resolution of identity approximation (RIJCOSX) in conjunction with the def2/J auxiliary basis.

For the pristine clusters (C_*n*_), the ground state is a singlet (S = 0) and only singlet–singlet transitions were considered, using the default restricted Kohn–Sham (RKS) formalism for closed-shell systems. For the nitrogen-doped clusters (C_*n*–1_N), the ground state is a doublet (S = 1/2), and TD-DFT was performed in the unrestricted framework, yielding spin-conserving doublet excitations. Solvent effects were included in the spectra calculation using the CPCM model^53^ with the parameters for water. Excited states were computed, requesting up to 20 roots. The complete workflow is illustrated in Figure 1.

**Figure 1:**
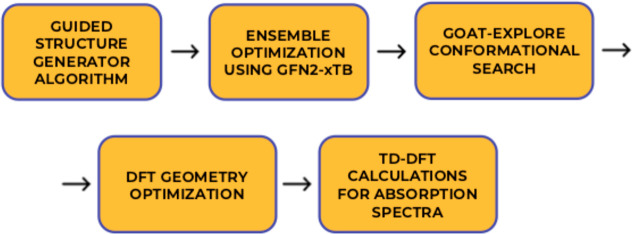
Scheme of the computation protocol applied in this work.

A sensitivity test was conducted for one representative cluster for both classes: C_20_ and C_19_N, and is discussed in Section S1. The spectra from the hybrid functionals ωB97X^54^ and CAM-B3LYP^56^ were compared to double hybrid functionals (thus, including double corrections to excited state energies), specifically ωB2PLYP^55^ for C_20_ and SOS-ωPBEPP86 ^57^ for C_19_N. For both clusters, the overall position of the absorption and the ordering of the main transitions were remarkably consistent among ωB97X and the higher level functionals. Bigger discrepancies were noted for intensities in the open shell system, as expected.

Overall, ωB97X was selected as the production functional for all spectra because it provides the best compromise between accuracy and computational efficiency. The spectra are reported in Figures S1 and S2. Additional validation is provided in Figure S3, where we present a comparison between the computed spectra of fullerene at the ωB97X&Def2-TZVP level with experimental data.

All spectra in this work were convoluted using the same Gaussian broadening to ensure a consistent comparison of spectral shapes and intensities, applying a constant full width at half-maximum (fwhm) of 0.12 eV to each transition.

## 3 Discussion and Results

### 3.1 Structural Analysis

Exemplary structures chosen among the lowest-lying ones of each ensemble are reported in Figures 2 and 3. Some of these structures (for *n* < 35) have already been presented and commented on in our previous work.^40^ Here, we extend the dimension of the cluster from 35 to 60,the latter being the size where the symmetric, IPR-compliant fullerene emerges naturally as the unique and most stable structure. . For clarity, in Figures 2 and 3, we have reported only the structures with an even number of atoms, while the odd-numbered counterparts are shown in Figure S4 of the Supporting Information. All structures reported here are hydrogen-free and represent unsaturated CCs; hydrogen-terminated PAHs are intentionally excluded from the present model.

**Figure 2:**
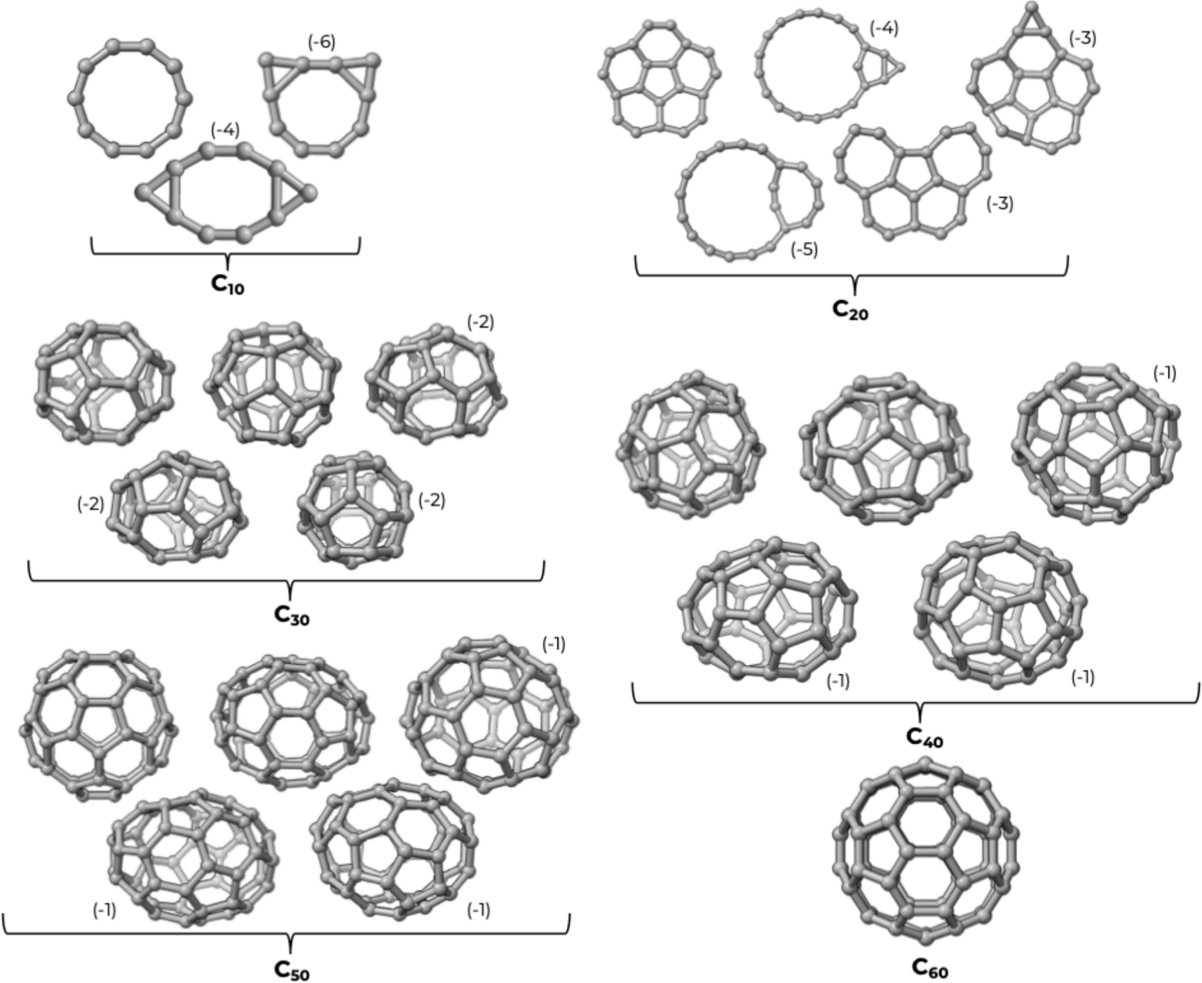
Three-dimensional structures for the pure C_*n*_ clusters. The negative numbers in parentheses indicate the energetic destabilization in terms of atomization energies in kcal/mol with respect to the most stable isomer. Where the difference in atomization energy was less than 1 kcal/mol, we omitted the energy indicator.

**Figure 3:**
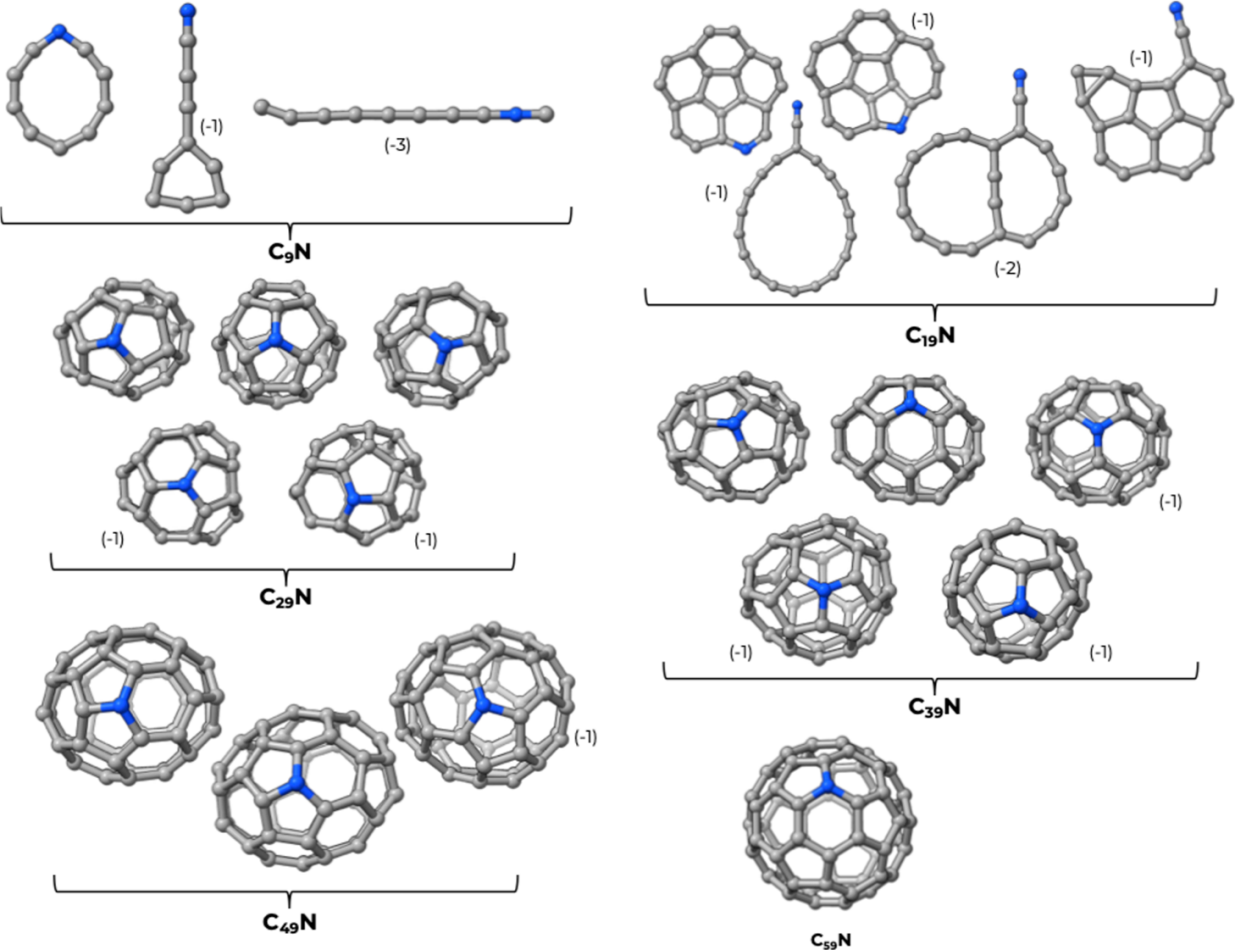
Three-dimensional structures for the doped C_*n*–1_N clusters. The negative numbers in parentheses indicate the energetic destabilization in terms of atomization energies per atom in kcal/mol with respect to the most stable isomer. Where the difference in atomization energy was less than 1 kcal/mol, we omitted the energy indicator.

The structures that we found for the neat cluster family are in line with previous (recent) calculations.^58–61^ In particular, the structures of C_10_, C_20_, and C_25_ are the same as those reported by ref 58, and the larger ones match those reported in ref 61. Our optimized structures occasionally differ from those reported in the literature. This discrepancy arises both from methodological differences and from the coexistence of several low-lying isomers, which DFT methods are often unable to discriminate univocally. In addition, the isomers correspond to local minima are separated by very high energy barriers, a situation that hinders an exhaustive exploration of the corresponding potential energy surface. As discussed in ref 61, through an analysis of past calculations, a significant variability of the structural data and corresponding energetic ordering for these clusters is expected.

One case where we found a striking difference with previous data is the C_30_ cluster. Our procedure converged on a cage structure that contains one 4-member carbon ring. By purposely transforming this structure into the standard one with only 5- and 6-membered rings, we confirmed that (at least at our calculation level) the one with the 4-member ring is more stable than the standard one of ∼5 kcal/mol. The geometries of the 6 lowest-lying isomers of C_30_, including the standard one, are reported in the Supporting Information in Figure S5 along with their relative energy.

Returning to Figures 2 and 3, we see that, as the cluster size increases, both families exhibit a structural evolution from ring-like configurations to planar (aromatic-like) forms and eventually to cage-like architectures reminiscent of fullerenes.^58,61^

Apart from the smaller members of the two families, the difference in atomization energy (see below) between the reported isomers is small and often less than 1 kcal/mol. Hence, species such as C_40_ or C_39_N are expected to display a variety of slightly different spatial arrangements whose difference lies in the shape and nature of the rings of the cage.^62^ Despite having essentially the same energy, these isomers are not easy to interconvert due to the extremely high barrier between them and the complex pathway along the transformation that requires breaking the cage and passing through more disordered intermediates. In other words, the ensemble of possible structures, of which we present only a few elements, is not in thermal equilibrium at any reasonable temperature in which CNDs exist. Owing to their intrinsic rigidity, these motifs are likely to be kinetically frozen and unable to interconvert under typical conditions. Although some larger clusters adopt cage-like geometries, these structures are generally distorted and variably strained and should not be interpreted as ideal fullerenes, but as limited-size models of kinetically trapped structural motifs embedded within an amorphous carbon matrix.

We can therefore assume that, within CNDs, such structures might correspond to amorphous and unsaturated carbon domains, possibly reflecting residual species generated during the synthetic process. Notably, they are found to be nearly equivalent in atomization energy, suggesting that several distinct local minima may coexist within the amorphous regions of the carbon framework.

This concept is further described in Figure 4 where we report the atomization energy for each cluster along the two families as a function of the number of atoms. The atomization energy is calculated using the following equation, where we use the isolated electronic energies of the carbon and nitrogen atoms in their ground state.

**Figure 4:**
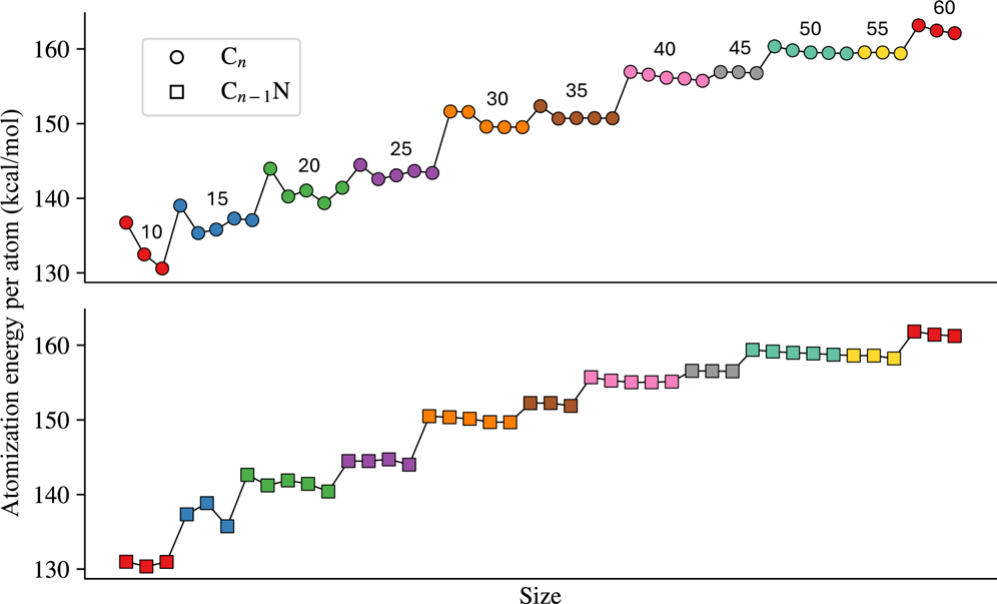
Atomization energies for C_*n*_ (top) and C_*n*–1_N (bottom). Each value of *n* is identified by a different color consistently across the classes. The correspondence between color and *n* is explicitly reported in the top panel.


$$E_{at}=\frac{\sum_{i}^{n}E_{i}(atom)-E(cluster)}{n}$$

This definition allows comparison of the resulting energies across clusters of different sizes. The larger the value, the higher the energy required to dissociate it into atoms, hence the stability of the cluster itself.

The stability is between 130 and 160 kcal/mol per atom and grows essentially linearly with cluster size, thereby pointing to the existence of strong many–body interactions well beyond 2-body and 3-body terms. This value is very large, typical of strongly bound clusters (e.g., metals), and is an indicator of how difficult it is to modify the structures in the absence of energetic processes. Also, the shape of the data in Figure 4 shows the uniformity of the stability across the isomers of a specific size. Again, this might indicate that the unsaturated amorphous carbon domain might be the result of the formation of several similar and isoenergetic coexisting isomers.

In Figure 5, we report an analysis of the hybridization pattern along the two families of clusters. In the same figure, we also report the average C–C bond distances. For both hybridization and distances, we have reported their averaged values over isomers of the same size.

**Figure 5:**
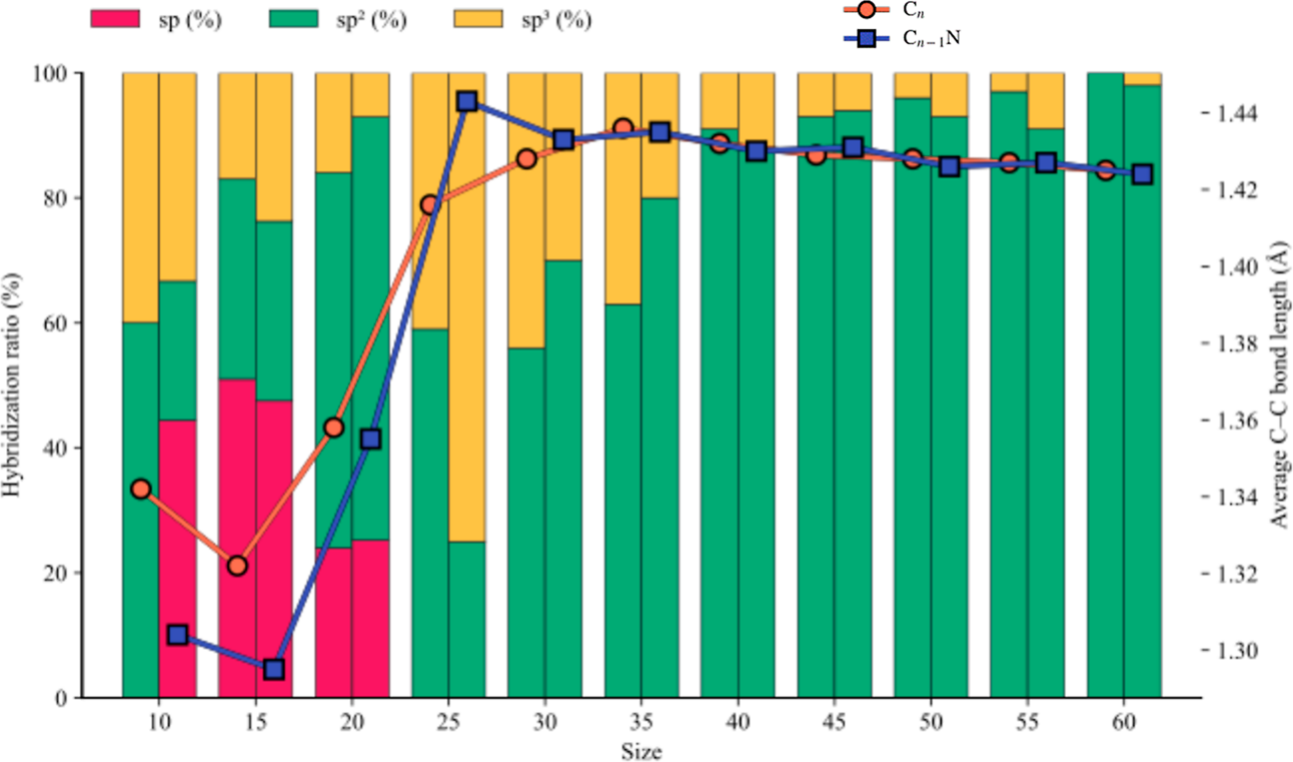
Hybridization patterns (%) of the carbon atoms in each cluster of the two families. The C_*n*_ are reported on the left bar and the C_*n*–1_N on the right one. The hybridization pattern averaged over the isomers of the same size. Average C–C bond lengths are reported superimposed on the histogram. In orange, the pure clusters and in blue, the doped ones. The *x*-axis refers to the total number of atoms in the clusters.

The fraction of sp^2^-hybridized carbon atoms increases as the average C–C bond distance approaches approximately 1.40 Å, while a higher sp^3^ contribution corresponds to longer bond lengths, typically around 1.53–1.55 Å. Clusters with shorter C–C bonds indicate a greater degree of π conjugation and planar character. When a cluster exhibits a significant sp contribution, its mean C–C distances shift toward shorter values (around 1.20 Å), reflecting the formation of cumulene- or acetylene-like linkages. Clusters whose size is greater than 25 atoms are dominated by sp^2^ and sp^3^ environments.

The introduction of a nitrogen atom into the carbon framework generally leads to a significant increase in the sp fraction for C_9_N and a moderate increase in the sp^2^ linkages for *n* = 30 and 35. These variations are connected, in turn, to a corresponding significant decrease (*n* = 10, 15, and 20) or increase (*n* = 25) of the average C–C distance. For larger clusters, the effect of doping is comparatively diluted in terms of geometric structure. This trend is consistent with nitrogen promoting local π conjugation and stabilizing linear and cage-like configurations, particularly when it replaces a carbon atom in a 6- or 5-membered ring. Clusters with higher sp^3^ content are expected to display greater deviations from planarity due to local pyramidalization. This structural distortion is evident in C_20_ and C_25_ and their corresponding N-doped versions.

As we have already mentioned, while the neat clusters were considered in their singlet state, the nitrogen-doped ones are in a doublet state. For C_*n*–1_N, we have mapped the spin density of each structure, and we have reported examples of it in Figure 6 for the even-numbered species. We have noticed that despite N being the source of spin unpairing, the spin density is not localized on nitrogen. In the small C_9_N, the spin polarization delocalizes over the entire structure and shows alternate regions of negative and positive spin polarization along the ring. Already at *n* = 20 (C_19_N), however, the spin polarization localizes on one side of the structure, where it is more prominent on the carbon atoms that are adjacent to the nitrogen. At *n* = 30 (C_29_N), we have a transition to a closed cage structure. The relatively small dimension of the cage still allows for a fairly marked delocalization of the spin polarization on the side far from the nitrogen. The same pattern, but with a more pronounced localization, is found for larger cages such as C_49_N and C_59_N.

**Figure 6:**
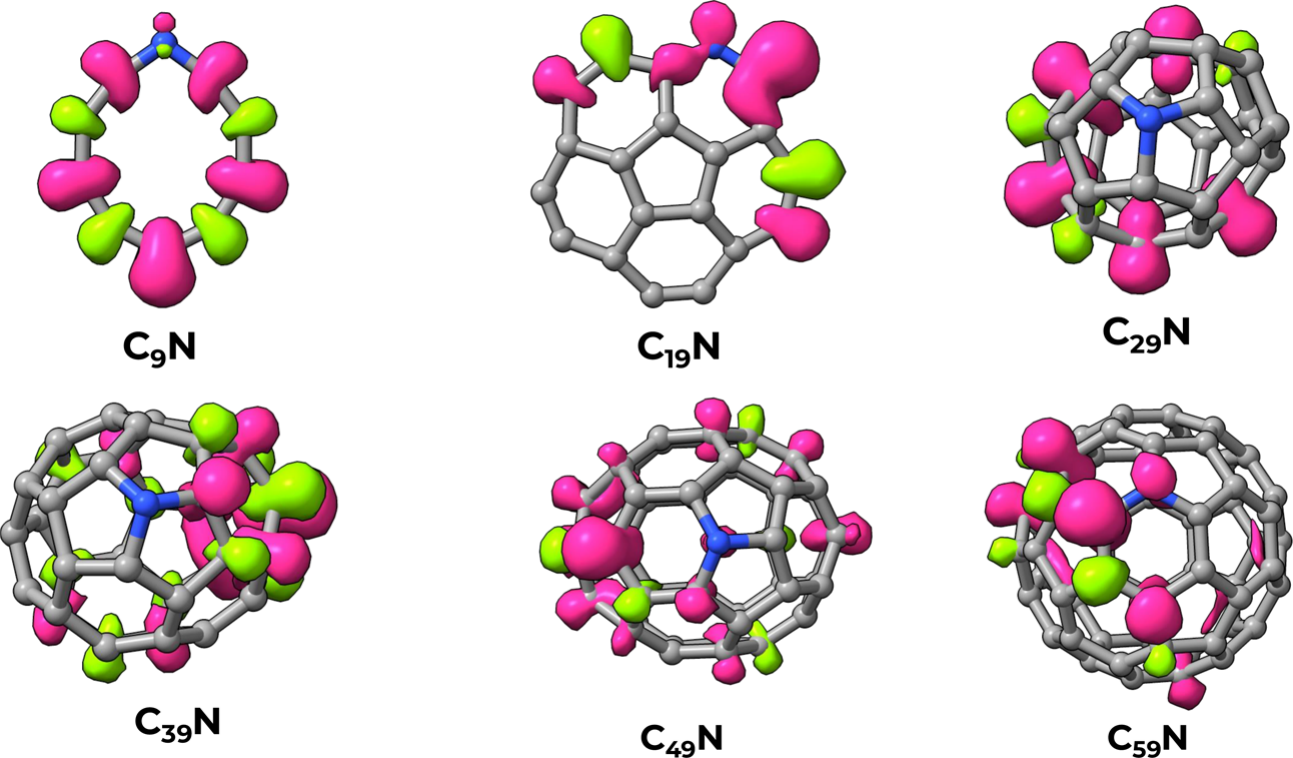
Spin-density maps for the nitrogen-doped clusters: the negative region is colored in lime green, the positive one in pink.

This behavior seems to be consistent with what is known for extended graphenic systems, where substitutional (graphitic) nitrogen incorporation in the graphene lattice leads to *n*-type doping, as the additional electron from nitrogen populates the delocalized π manifold. Consequently, the spin polarization is quenched, and the unpaired spin density is slightly delocalized over neighboring carbon atoms.^63^ Here, however, the lack of an electronic continuous band due to the finite size of the systems still allows for a local excess of the spin polarization.

### 3.2 Absorption Spectra

Absorption spectra for a selected number of clusters (*n* = 10, 20, 30, 40, 50, and 60) are shown in Figure 7. The absorption spectrum of neat C_*n*_ is in black and the one for doped C_*n*–1_N in red. In this discussion, we shall focus on the visible and near UV range between 250 and 750 nm. The absorption of N-doped clusters is generally weaker and has been scaled appropriately to make the comparison easier. In each panel of Figure 7, on top, we report the structure of the specific isomer for which the spectra is shown. The data pertaining to the most intense transitions (excited state, wavelength, and oscillator strength) are reported in Table 1. The pure carbon data, although here shown for a single structure and not for the entire ensemble (see also Section 3.2.2 for an assessment of ensemble effects), roughly match the estimates based on the scaled semiempirical approach of ref 60, where densely populated ensembles were used. The spectra presented there show the onset of absorption around 400–300 nm and a first intense peak at 220 nm, similar to ours.

**Figure 7:**
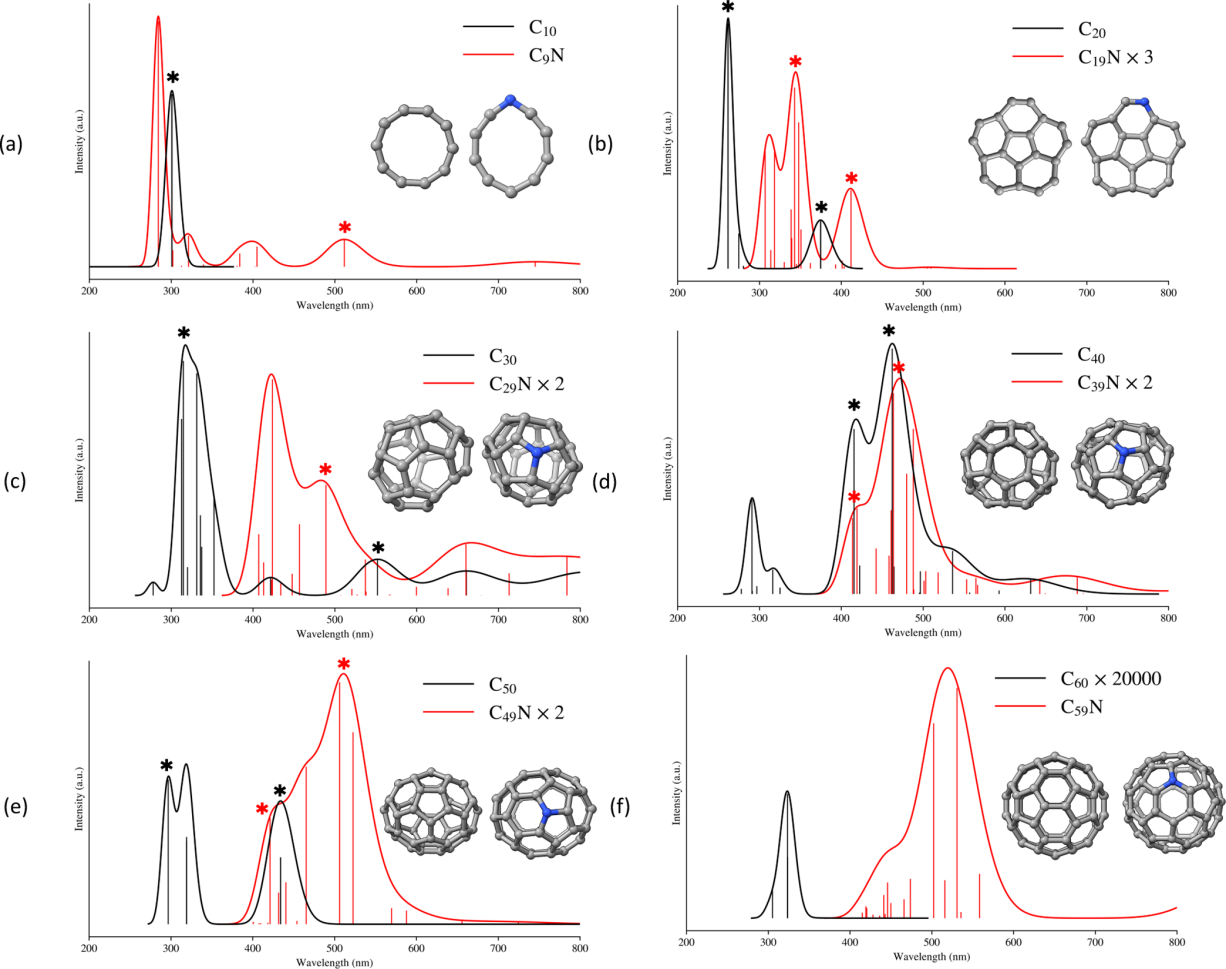
Comparison between the absorption spectra for C_*n*_ (in black) and their corresponding-doped structures C_*n*–1_N (in red): (a) *n* = 10, (b) *n* = 20, (c) *n* = 30, (d) *n* = 40, (e) *n* = 50, and (f) *n* = 60. On top of each panel, we report the cluster structures. Some spectra are scaled to improve readability. The transitions marked with * have been selected for the density difference analysis (see Figure 8).

**Table 1:** Absorption Line Positions (nm) Calculated at the ωB97X/def2-TZVP Level for the Lowest-Energy Isomer of Selected Clusters^a^

cluster	state	nm	*f*	state	nm	*f*
C9N	1	744.9	0.0022	**2**	**511.6**	**0.0122**
	6	404.9	0.0089	8	383.8	0.0058
	11	339.5	0.0010	12	321.2	0.0137
	15	302.0	0.0073	17	284.3	0.0023
	18	284.2	0.1089			
C10	9	301.0	0.0393	**10**	**300.8**	**0.0393**
C19N	**5**	**411.9**	**0.0039**	12	348.0	0.0034
	**14**	**343.0**	**0.0042**	16	338.6	0.0013
	18	318.3	0.0036	20	306.9	0.0045
C20	**1**	**374.8**	**0.0038**	2	374.7	0.0037
	15	274.7	0.0027	17	261.6	0.0194
	**18**	**261.6**	**0.0195**			
C29N	1	877.6	0.0043	2	783.6	0.0064
	3	713.0	0.0035	4	660.3	0.0091
	5	638.4	0.0011	6	599.7	0.0013
	9	537.3	0.0057	11	520.8	0.0010
	**13**	**489.1**	**0.0218**	14	456.8	0.0114
	15	447.8	0.0034	16	433.9	0.0019
	17	423.8	0.0348	18	422.6	0.0025
	19	412.9	0.0053	20	407.0	0.0098
C30	1	810.6	0.0107	4	660.8	0.0111
	**6**	**552.2**	**0.0166**	7	421.5	0.0082
	10	352.3	0.0306	12	337.4	0.0114
	13	335.7	0.0188	**14**	**331.3**	**0.0576**
	16	319.9	0.0066	17	314.8	0.0550
	18	312.7	0.0414	20	278.0	0.0060
C39N	3	688.5	0.0017	9	518.4	0.0011
	10	503.3	0.0011	13	487.9	0.0083
	14	479.9	0.0061	**15**	**463.6**	**0.0107**
	16	460.9	0.0042	17	458.3	0.0019
	18	442.6	0.0023	**19**	**419.3**	**0.0063**
	20	413.7	0.0038			
C40	1	631.4	0.0034	4	536.0	0.0099
	5	496.6	0.0051	8	464.5	0.0063
	**9**	**462.2**	**0.0562**	11	422.4	0.0065
	**12**	**415.6**	**0.0378**	13	325.1	0.0014
	14	316.2	0.0055	16	296.8	0.0018
	18	290.8	0.0225	20	277.8	0.0011
C49N	4	587.9	0.0026	6	569.7	0.0031
	10	522.6	0.0372	**11**	**506.1**	**0.0476**
	12	465.3	0.0412	14	440.5	0.0081
	15	431.7	0.0061	**16**	**421.2**	**0.0251**
C50	**8**	**434.0**	**0.0407**	9	434.0	0.0407
	16	319.1	0.0530	17	319.1	0.0507
	**20**	**296.6**	**0.0955**			

a The data in bold pertain to those excitations whose density maps are in Figures 7 and 8. We have reported only those transitions with oscillator strengths (*f*) larger than 10^–3^.

We begin our analysis with the simplest system that is C_10_. The cluster structure is essentially a planar ring. Its main absorption band (Figure 7a) is well localized at 300 nm. Only two bright states contribute to this band, S_9_ and S_10_, which are almost degenerate with an energy difference of 3 meV. The two transitions have the same compositions in terms of single excitations. The dominant ones are the H → L (28%)[Fn fn1], the H-1 → L+1 (28%), followed by two excitations with a weight of 22% (H-2 → L+2 and H-3 → L+3).

Due to its open shell nature, the corresponding C_9_N clusters shows several additional low-energy absorption bands at high wavelengths (see Table 1 for details). The first one at 745 nm is essentially due to a H_α_ → L_α_ single excitation. The second band falls at 512 nm and is due to a H_β_ → L_β_ excitation (85%). The two transitions at 404 and 384 nm are mixed, due to several excitations, but dominated by the (S-3)_α_ → L_α_ (56%) and (S-1)_α_ → (L+2)_α_ (56%), respectively. The brightest band at 284 nm corresponds to a transition to S_18_ and, as expected, is due to a heavily mixed set of excitations that includes transitions both within the α and β manifolds.

The density difference maps for two illustrative selected transitions for C_10_ and C_9_N are reported in Figure 8. In C_10_ (S_0_ → S_10_), due to the symmetric structure, the electron density is depleted (blue regions) inside the ring and moved onto the edge (red regions) with no or very little charge transfer across the molecular structure. Doping with nitrogen introduces an asymmetry that enhances charge transfer across the ring in both transitions. For example, for the low wavelength one (S_0_ → S_2_), a significant portion of the electron density is transferred from the carbon atoms adjacent to the nitrogen atom toward the carbon atom on the opposite side of the ring.

**Figure 8:**
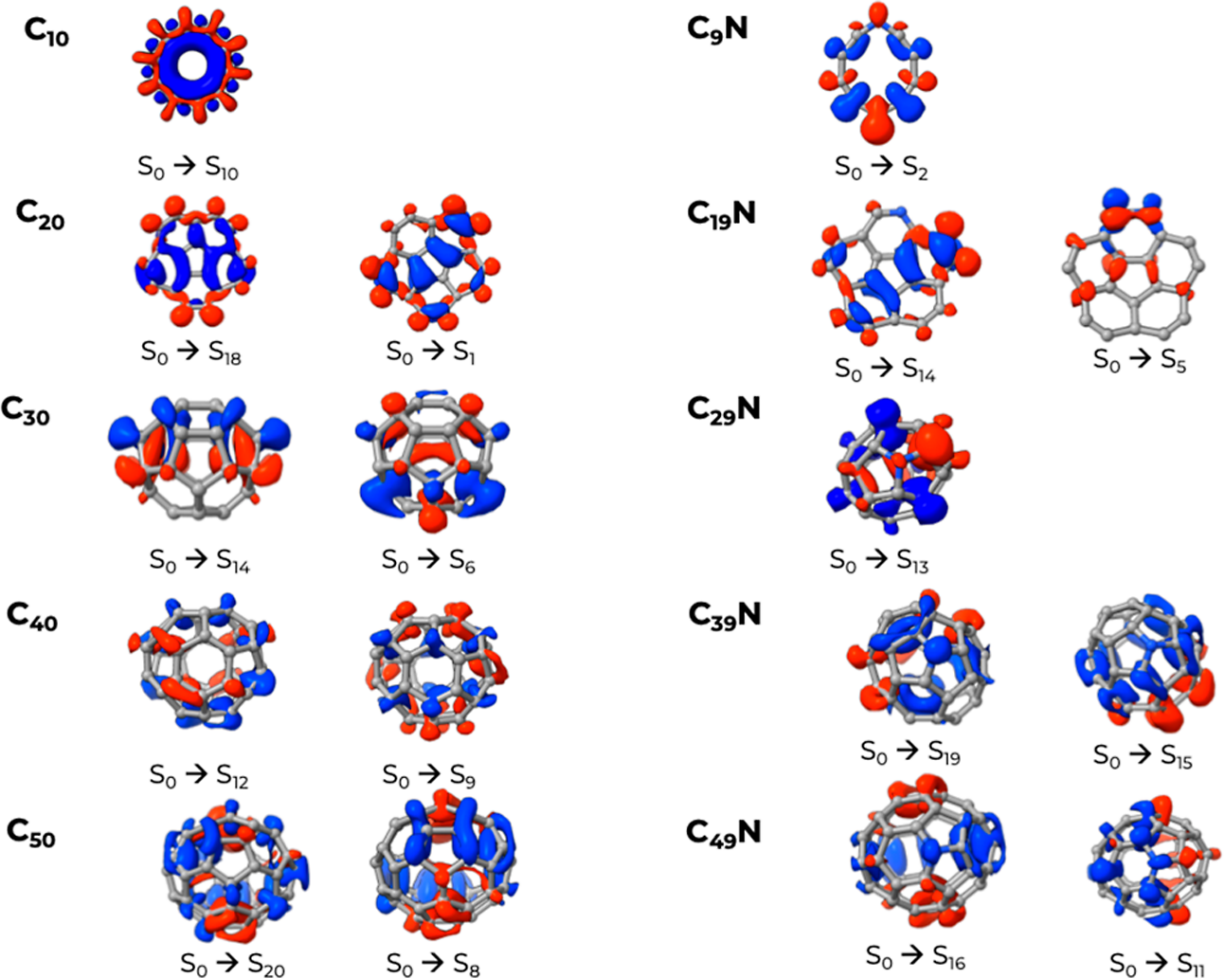
Density difference maps for the indicated transitions in the two families of clusters. The regions in blue correspond to electron depletion, and the red ones to electron accumulation. The corresponding bands in the spectra are indicated in Figure 7 by an asterisk.

C_20_, most stable isomer, has a nearly planar structure. The cluster has two main absorption bands (375 and 262 nm, see Figure 7b and Table 1). Both are due to transitions to nearly degenerate pairs of electronic states, S1 and S2 for the former and S17 and S18 for the latter. The excitations are heavily mixed and reflect the strongly multideterminantal nature of amorphous CC. From the density differences in Figure 8, we see that the excitations are delocalized over the entire structure, and the trend (as in C_10_, but less symmetric) is that of shifting density from the center of the molecule to its edges. The absorption pattern of C_19_N (Figure 7b) shows that the first two absorption bands are significantly red-shifted with respect to its neat counterpart, with former at 412 nm (entirely due to the S_0_ → S_5_ transition) while the second, in the region between 300 and 350 nm, involves many transitions to several highly excited states. All transitions are heavily mixed, but the one at 412 nm has a significant contribution from Hα → Lα excitation. At difference with C_20_, the electronic transitions are more localized on specific regions of the molecule. The S_0_ → S_14_ shows a significant character of charge transfer from the central 5-member ring toward the edge of the structure.

C_30_ and C_29_N are examples of clusters where the cage structure dominates over other isomers. The absorption spectra are in Figure 7c. The presence of an asymmetric cage in C_30_ induces the appearance of several high-wavelength transitions corresponding to low-lying excited states. The transition to first excited state S_1_ falls at 811 nm followed by S_4_ and S_6_ at 661 and 552 nm, respectively. Each of these three transitions is dominated by a single excitation, namely, H → L for S_1_, H-1 → L for S_4_, and H-4 → L for S_6_. The density difference map for S_0_ → S_6_ is shown in Figure 8 and it involves a redistribution of the electron density over the cage but in an alternating pattern of depletion and accumulation. At shorter wavelengths, C_30_ has a more active absorption due to many excited states that emerge from the combination of several singly excited configurations. For example, the line at 331 nm corresponding to S_14_ is due to H-2 → L+1 and H-4 → L+1. The overall density difference for this transition is shown in Figure 8, and involves a certain degree of charge transfer from the polar region of the cage to the equatorial one. When moving to the doped variant, C_29_N, one can see that the geometry undergoes a significant distortion. The number of 6-member rings decreases from 5 in C_30_ to 4 in C_29_N, leading to a more strained structure. Consequently, the transitions are distributed over a larger energy range. For C_29_N also, the intense absorption band is significantly red-shifted with respect to the neat cluster. The excitation pattern is made by several transitions whose origin is difficult to trace back to well-defined single excitations. The density analysis in Figure 8 for the S_0_ → S_13_ shows how it has charge transfer character and electronic density shifts toward the carbon adjacent to nitrogen in a way that is reminiscent of what we have already seen for C_9_N.

The C_40_ and C_39_N species are unique in our selected sets of structures. The absorption of the N-doped cluster is not as strongly red-shifted as in all other cases. This is likely due to the similarity of the two structures. It is known^62^ that C_40_, among the small fullerenes, is particularly stable despite still not being big enough to satisfy the IPR rule (the first one to do it is C_60_). The nitrogen substitution for the specific isomers that we have found does not alter the overall cage structure, maintaining the same pattern of 10 six-membered and 12 five-membered rings. As for C_30_, the electronic excitations lead to intense absorptions only when highly excited states are involved (see Figure 7d). Also, as seen before, the transitions in the neat variant are delocalized over the entire cage structure (Figure 8) while having a more significant character of charge transfer in the N-doped one. Particularly indicative is the case of S_0_ → S_15_, where the density indicates a shift of negative charges on the portion of the cage opposite to the nitrogen atom (Figure 8, from blue regions to red ones).

The last cluster that we have characterized are C_50_ and C_49_N. In this case, the cage is less strained owing to its topological proximity to C_60_. Both cages are composed of 12 five-membered and 15 six-membered rings, but their overall shape is different, with the doped cluster being more spherical. The absorption pattern is similar to the one we have seen for the previous structures. The N-doping induces a significant redshift of the main absorption band. The individual transitions in C_49_N have charge transfer character.

Figure 9 essentially summarizes the results we have discussed so far. The absorption spectra of the two families of clusters are reported on the same scale and as a function of the number of atoms. The data on the left shows clearly how the absorption in the C_*n*_ family is strongly dependent upon the geometry of the chosen isomer.

**Figure 9:**
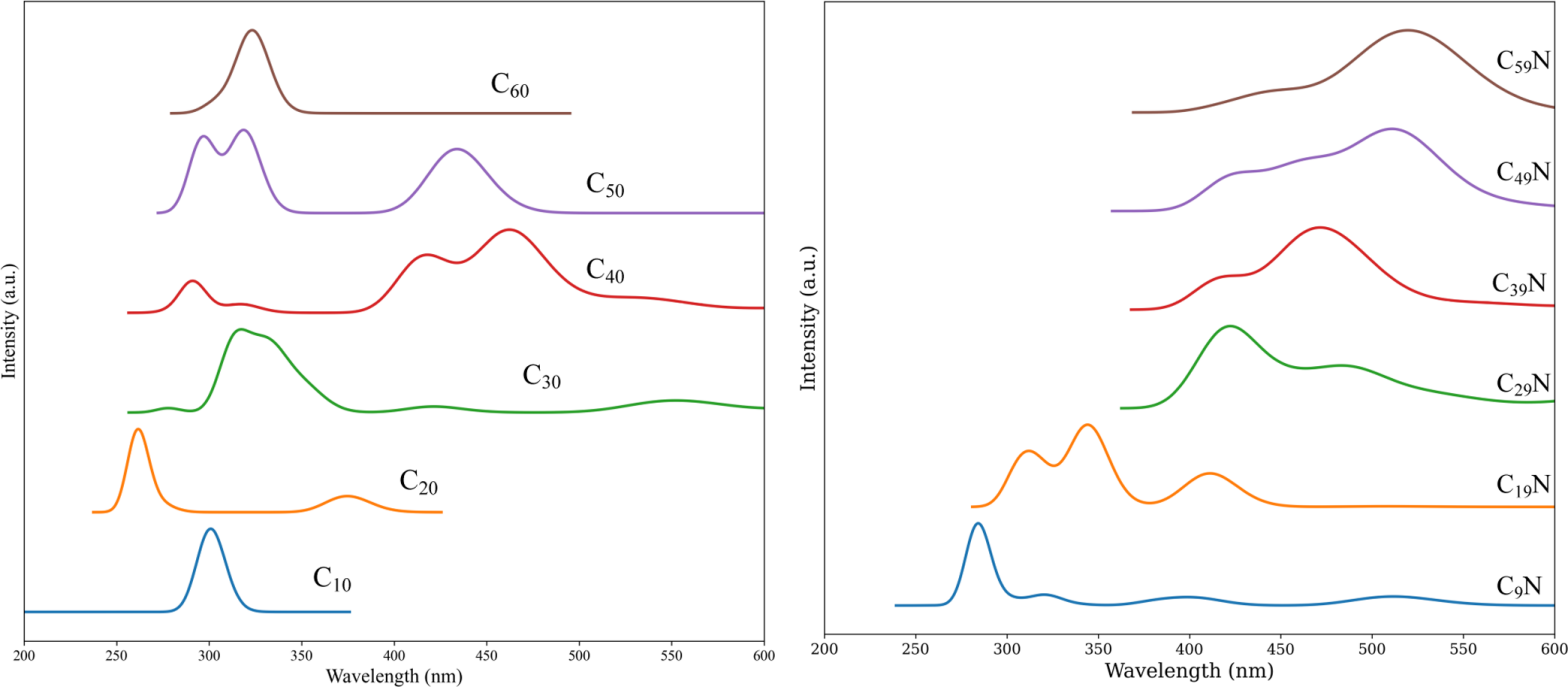
TD-DFT calculated absorption spectra for pure CCs (on the left) and nitrogen-doped ones (on the right), normalized and vertically shifted. From bottom (smallest) to top (largest), the spectra correspond to increasing cluster size.

The more regular the geometry (C_10_ and C_60_), the more compact the absorption region. When the structure is richer in sp^2^ content and tends toward planar (C_20_), the absorption shifts to higher energies, an effect already noticed in ref 60. Irregular cages (C_40_ and C_50_) show a red-shifted absorption pattern, inducing the existence of transitions to low-lying excited states with high transition moments.

The general trend in the C_*n*–1_N family is more evident. Cluster growth leads to an increased redshift of the first bright absorption band due to the progressive increase in transition moment for low-energy transitions involving the H → L (and adjacent orbitals) excitations in the two spin manifolds. It seems that upon N doping, a redshift appears because of changes in the electronic structure. In the doped series, this redshift further increases with cluster size, even for single-atom substitutions. This size dependence is absent in the neat clusters.

#### 3.2.1 Geometric Variations at High Energy

As stated in the Methods section, for each cluster composition, a search for local minima was conducted using GOAT driven by the GFN2-xTB semiempirical energy. Only the low-energy isomers of these ensembles of structures were then further optimized at the DFT level and the spectra calculated. We spend here few words on the high energy range of structures as obtained from the semiempirical calculations. We begin from the pure CCs.

From C_10_ to C_20_, the high-energy structures display a pronounced structural diversity (analogous to those reported in Figure 2). Three main motifs can be identified. The first consists of planar or nearly planar PAH-like structures, characterized by fused-ring arrangements and predominantly sp^2^ carbon coordination, with only minor out-of-plane distortions. The second motif includes structures dominated by one or a few very large carbon rings that remain mostly planar but are very extended with respect to compact fused-ring systems. The third motif is represented by highly strained early cage-like geometries, in which the carbon framework already shows significant curvature and closure, indicating the possible onset of three-dimensional organization even at relatively small sizes. Hand-selected examples are reported in Figure S6.

As the cluster size increases, the relative population of these motifs changes abruptly. For clusters containing approximately 30 carbon atoms, the structural diversity observed at smaller sizes has disappeared. At this size, all candidates with energies below 60 kcal/mol adopt closed, cage-like configurations. These structures are compact, fully three-dimensional, reminiscent of fullerene-type morphologies, although not necessarily corresponding to high-symmetry fullerenes.

The situation is similar for the N-doped families of clusters. For the smallest systems, up to C_19_N, the low-energy structures exhibit a wide conformational diversity that closely mirrors, but also enriches, that of the pure CCs. Planar or nearly planar PAH-like structures remain common, with the nitrogen atom incorporated into the carbon framework within fused-ring motifs but preserving an overall planar topology. Large-ring structures are also observed, in which the nitrogen atom adopts often a peripheric position. In addition, half-cage conformers appear at small sizes. A selection of possible structures is in Figure S6.

As the cluster size increases, the population of planar, open, and curved motifs progressively shifts toward three-dimensional structures. For larger clusters, C_29_N and above, the structures within 60 kcal/mol are uniformly cage-like. Compared to the pure carbon case, nitrogen substitution tends to lower the symmetry of the cages and increase the number of accessible low-energy conformations, as the heteroatom breaks the equivalence of carbon sites, introducing structural diversity.

In conclusion, due to the prevalence of cage-like structures over a wide energy range, the selection of low-lying candidates done in the preceding sections appears fully justified, as the structural uniformity observed for clusters with *n* > 30 makes them suitable for the calculation of representative absorption spectra.

#### 3.2.2 Impact of Isomers on the Absorption Spectra

The computed spectra, as expected, depend on the geometry of the specific isomer, especially for the neat cluster families. The variation is evident when moving from cyclic-linear structures to planar ones and cages. However, even between seemingly very similar cages, the absorption spectra turned out to be very sensitive to the specific geometric features (relative abundance of 5- and 6-membered rings, for example) and to the overall geometric distortion and strain. To provide data that are less biased toward a specific choice, we have averaged the spectra over the lowest 5 isomers of each family of clusters with an even number of atoms. Because CCs often form through uncontrolled synthetic pathways, their final structure may correspond to any of several low-energy isomers, due to intrinsic rigidity that prevents interconversion and, ultimately, the existence of a thermally accessible ensemble of structures. Hence, we computed the simple averaged absorption profile of the five lowest isomers to account for the distribution of possible final structures and to obtain a spectrum that might represent any experimental outcome more realistically.

The results are reported in Figure S7 for the same choice of isomers as Figure 7. In all cases, except one, we clearly see that some of the peculiarities we have pointed out in the discussion of Figure 7 disappear, and the general trend is more discernible. In particular, all clusters show a significant redshift in their doped variant, with the main absorptions moving from 250 to 300 nm in the neat cluster to 300–500 nm.

#### 3.2.3 Low Lying States of Different Multiplicity

The CCs of the C_*n*_ family, as we have shown in ref 40, have HOMO–LUMO gaps that decrease when moving from linear and cyclic structures to cage-like ones. However, LUMO energies are only a qualitative indicator of electronic properties and are strongly dependent on the chosen functional/basis-set combination. In contrast, the energy of the first triplet state is far less method-dependent and describes essentially the same information, making it a more reliable descriptor, especially when it comes to photodynamic processes. The vertical energies of the first triplet state for the C_*n*_ family are shown in Figure 10 (yellow bars). This singlet–triplet separation is below 3 eV on all the candidate structures and, as expected, is the lowest (∼1 eV) for strained cages such as C_30_, C_40_, and C_50_.

**Figure 10:**
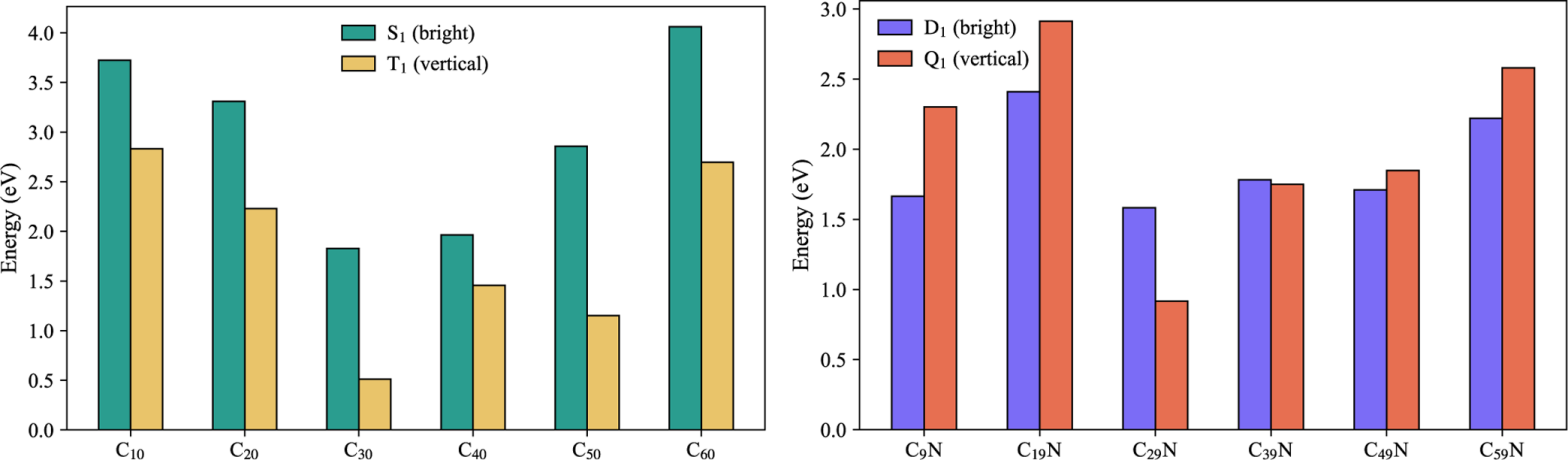
Comparison between the lowest bright spin-conserving transitions (TD-DFT/TDA) and the lowest spin-forbidden vertical gap (from ΔSCF-UKS) at the ground-state geometry for pure CCs (on the left) and doped ones (on the right). Calculations in water (CPCM, ε = 80.4, *n* = 1.33) with ωB97X-D3/def2-TZVP.

The cyclic (C_10_), planar (C_20_), and symmetric cage (C_60_) have the largest singlet–triplet gaps. In the same figure, we have also reported the first singlet excitation energy with a nonvanishing transition dipole moment (green bars). This might serve as an indicator of the likely state from where radiative decay can still occur after singlet–singlet interstate relaxation of high-lying, brighter states. As shown by Table 1, the lowest lying excited states of neat clusters have small or near-vanishing transition dipole moments. Hence, one can surmise that nonradiative decay due to internal conversion and intersystem crossing may become efficient and suppress fluorescence.

For the doped variants, the energy location of the first quartet state (red bars of Figure 10) is comparable to the triplet positions of the neat family. The comparison with the position of the first optically active doublet state (purple bars of Figure 10) shows clearly how, in many cases, the fluorescent state has a lower energy than the spin-forbidden one. Also, as shown in Table 1, the lowest-lying excited states of the C_*n*–1_N family have larger transition dipole moments than the pure clusters, thus making radiative decay comparatively more likely.

## 4 Conclusions

In this work, we have investigated a family of amorphous CCs (C_10_–C_60_) and their singly nitrogen-doped analogues as minimal models for the unsaturated domains of CNDs. By combining an extensive isomer search at the semiempirical level based on the GOAT algorithm with DFT and TD-DFT calculations, we explored how size, geometry, and local hybridization patterns shape the electronic structure and absorption properties of these systems.

Across the entire size range, both neat and doped clusters display a varied energetic surface characterized by multiple local minima. As cluster size increases, the dominant motifs evolve from rings and planar fragments toward more compact cage-like geometries. This geometric variability, combined with the rigidity of the emerging cages, suggests that amorphous regions in CND cores may consist of a frozen ensemble of structurally distinct but energetically comparable motifs—an aspect that is often overlooked in coarse-grained models of CNDs.

Nitrogen substitution perturbs these geometries in size-dependent ways: small clusters exhibit significant local rearrangements and increased sp or sp^2^ character, while larger cages retain their overall topology. Spin-density analyses showed that the unpaired electron introduced by nitrogen remains largely delocalized over the carbon framework, often far from the doping site, echoing the behavior observed in finite graphenic fragments but amplified by the finite nature of the clusters.

The computed absorption spectra highlight fundamental differences between the two families. Pure CCs show strongly geometry-dependent UV absorption, with bright transitions concentrated in the near-UV region and only weak low-energy bands. In contrast, nitrogen-doped clusters consistently display brighter low-energy transitions and a pronounced redshift of the first band, also in cases where the overall cage geometry is only modestly perturbed. This behavior arises from changes in electronic structure and from the nature of charge-transfer character in the excited states, induced by the nitrogen atom.

Finally, the analysis of spin-forbidden states shows that, although nitrogen doping increases oscillator strengths and enables partially allowed low-energy transitions, it also compresses the manifold of states with different spin multiplicities. The resulting proximity of active doublet levels to low-lying (dark) quartet states enhances spin mixing and may activate fast nonradiative intersystem crossing, potentially suppressing fluorescence. This interplay between enhanced radiative channels and competing nonradiative pathways may help rationalize why nitrogen doping in real CNDs modulates—but does not straightforwardly maximize—photoluminescence efficiency.

Overall, the present results are far from providing a full atomistic model of CNDs, but nevertheless, can help to disentangle intrinsic electronic-structure effects from more complex surface and environmental contributions. Isolating the role of heteroatom substitution in unsaturated carbon domains, while maintaining a high-level computational scheme, this study has shown that local structural motifs of these domains can influence the optical behavior of CNDs. Such an approach can be extended to other dopants, higher doping concentrations, and partially saturated structures, and therefore can be applied to more complete and realistic models of CNDs.

## Supplementary Material



## Footnotes

a For simplicity, in the rest of the text, we will indicate the HOMO with H, and the LUMO with L.

## Notes

Assessment of TD-DFT method, comparison with experimental absorption bands for C_60_, structures of C_*n*_ and C_*n*–1_N cluster for odd values of *n*, low-lying isomers of C_30_, high-energy isomers of C_20_ and C_19_N at the semiempirical level, and TD-DFT spectra averaged over the lowest-lying isomers (PDF)

## References

[1] Baqiya M. A., Purwandari E., Asih R., Astuti F., Nakajima H., Ibrahim A., Wong L. H., Darminto (2025). A Brief Review and Prospect of Amorphous
Carbon and Reduced Graphene Oxides Derived from Biomass as a Low Cost
and New Photovoltaic Cell. Appl. Surf. Sci..

[2] Barbero F., Destro E., Bellone A., Di Lorenzo L., Brunella V., Perrone G., Damin A., Fenoglio I. (2025). Hydrothermal
Carbonization Synthesis of Amorphous Carbon Nanoparticles (15–150
Nm) with Fine-Tuning of the Size, Bulk Order, and the Consequent Impact
on Antioxidant and Photothermal Properties. Nanoscale Adv..

[3] Jones A. P., Fanciullo L., Köhler M., Verstraete L., Guillet V., Bocchio M., Ysard N. (2013). The Evolution of Amorphous
Hydrocarbons in the ISM: Dust Modelling from a New Vantage Point. Astron. Astrophys..

[4] Nietiadi M. L., Valencia F., Gonzalez R. I., Bringa E. M., Urbassek H. M. (2020). Collisions
between Amorphous Carbon Nanoparticles: Phase Transformations. Astron. Astrophys..

[5] Gabrielle
Sutanto L., Sabilla S., Wardhana B. Y., Ramadani A., Sari A. P., Anjani Q. K., Basirun W. J., Amrillah T., Amalina I., Jiwanti P. K. (2024). Carbon Nanomaterials as Electrochemical
Sensors for Theophylline: A Review. RSC Adv..

[6] Quílez-Bermejo J., Morallón E., Cazorla-Amorós D., Celzard A., Fierro V. (2025). Progress and
Perspectives in the Electrochemical Synthesis
of Carbon Nanomaterials. Carbon.

[7] Li H., Kang Z., Liu Y., Lee S.-T. (2012). Carbon Nanodots:
Synthesis, Properties and Applications. J. Mater.
Chem..

[8] Xu X., Ray R., Gu Y., Ploehn H. J., Gearheart L., Raker K., Scrivens W. A. (2004). Electrophoretic
Analysis and Purification
of Fluorescent Single-Walled Carbon Nanotube Fragments. J. Am. Chem. Soc..

[9] Zhu P., Wang S., Zhang Y., Li Y., Liu Y., Li W., Wang Y., Yan X., Luo D. (2022). Carbon Dots in Biomedicine:
A Review. ACS Appl. Bio Mater..

[10] Sturabotti E., Camilli A., Leonelli F., Vetica F. (2024). Carbon Dots as Bioactive
Antifungal Nanomaterials. ChemMedChem.

[11] Salvi A., Kharbanda S., Thakur P., Shandilya M., Thakur A. (2024). Biomedical Application
of Carbon Quantum Dots: A Review. Carbon Trends.

[12] Sturabotti E., Camilli A., Georgian Moldoveanu V., Bonincontro G., Simonetti G., Valletta A., Serangeli I., Miranda E., Amato F., Giacomo Marrani A., Migneco L. M., Sennato S., Simonis B., Vetica F., Leonelli F. (2024). Targeting the Antifungal Activity of Carbon Dots against *Candida Albicans* Biofilm Formation by Tailoring Their Surface
Functional Groups. Chem—Eur. J..

[13] Sargueil J., Santoni M., Cichella S., Sabato A. D., Moldoveanu V. G., Sturabotti E., Simonis B., Giustini M., Migneco L. M., Leonelli F., Vetica F. (2025). Carbon Dots as Nano-Photocatalysts:
A Green Tool for Indole and Heteroarenes Alkylation. Chem—Eur. J..

[14] Di
Sabato A., Santoni M., Moldoveanu V. G., Camilli A., Feroci M., Olivo G., Simonis B., Sturabotti E., Leonelli F., Vetica F. (2025). Synergistic Visible
Light Nanophotocatalysis and Asymmetric Organocatalysis for the Stereoselective
α-Alkylation of Aldehydes. Adv. Synth.
Catal..

[15] Maria
Vitagliano C., Camilli A., Georgian Moldoveanu V., Di Sabato A., Feroci M., Sturabotti E., Scognamiglio V., Leonelli F., Masi A., Vetica F. (2024). Selective
Interaction of Chiral Carbon Dots with Nucleic Acids: A Promising
Nanosensing Platform. Chem—Eur. J..

[16] Rosso C., Filippini G., Prato M. (2020). Carbon Dots as Nano-Organocatalysts
for Synthetic Applications. ACS Catal..

[17] Chen B. B., Liu M. L., Huang C. Z. (2020). Carbon
Dot-Based Composites for Catalytic
Applications. Green Chem..

[18] Goyal S., Chaudhary S., Umar A., Ibrahim A. A. (2025). Carbon Dots in Catalysis:
Synthesis, Properties, Applications, Comparative Advantages, and Future
Directions. J. Environ. Chem. Eng..

[19] Yu Y., Zeng Q., Tao S., Xia C., Liu C., Liu P., Yang B. (2023). Carbon Dots Based Photoinduced
Reactions: Advances
and Perspective. Adv. Sci..

[20] Paul, A. ; Kurian, M. Catalytic Applications of Carbon Dots. In Carbon Dots in Analytical Chemistry; Elsevier, 2023; pp 337–344.

[21] Ozyurt D., Kobaisi M. A., Hocking R. K., Fox B. (2023). Properties,
Synthesis,
and Applications of Carbon Dots: A Review. Carbon
Trends.

[22] Zhao X., Wei J., Song T., Wang Z., Yang D., Zhang X., Huo F., Zhang Y., Xiong H.-M. (2024). Computational Insights into Carbon
Dots: Evolution of Structural Models and Structure–Activity
Relationships. Chem. Eng. J..

[23] Mintz K. J., Bartoli M., Rovere M., Zhou Y., Hettiarachchi S. D., Paudyal S., Chen J., Domena J. B., Liyanage P. Y., Sampson R., Khadka D., Pandey R. R., Huang S., Chusuei C. C., Tagliaferro A., Leblanc R. M. (2021). A Deep Investigation
into the Structure of Carbon Dots. Carbon.

[24] Mocci F., de Villiers Engelbrecht L., Olla C., Cappai A., Casula M. F., Melis C., Stagi L., Laaksonen A., Carbonaro C. M. (2022). Carbon
Nanodots from an In Silico Perspective. Chem.
Rev..

[25] Shi B., Nachtigallová D., Aquino A. J. A., Machado F. B. C., Lischka H. (2019). Excited States
and Excitonic Interactions in Prototypic Polycyclic Aromatic Hydrocarbon
Dimers as Models for Graphitic Interactions in Carbon Dots. Phys. Chem. Chem. Phys..

[26] Shi B., Nachtigallová D., Aquino A. J. A., Machado F. B. C., Lischka H. (2019). High-Level
Theoretical Benchmark Investigations of the UV-Vis Absorption Spectra
of Paradigmatic Polycyclic Aromatic Hydrocarbons as Models for Graphene
Quantum Dots. J. Chem. Phys..

[27] Boukhvalov D. W., Osipov V. Yu., Hogan B. T., Baldycheva A. (2023). A Comprehensive
Model of Nitrogen-Free Ordered Carbon Quantum Dots. Discov. Nano..

[28] Margraf J. T., Strauss V., Guldi D. M., Clark T. (2015). The Electronic Structure
of Amorphous Carbon Nanodots. J. Phys. Chem.
B.

[29] Sinitsa A. S., Lebedeva I. V., Popov A. M., Knizhnik A. A. (2017). Transformation of
Amorphous Carbon Clusters to Fullerenes. J.
Phys. Chem. C.

[30] Strauss V., Margraf J. T., Dolle C., Butz B., Nacken T. J., Walter J., Bauer W., Peukert W., Spiecker E., Clark T., Guldi D. M. (2014). Carbon Nanodots:
Toward a Comprehensive
Understanding of Their Photoluminescence. J.
Am. Chem. Soc..

[31] Ai L., Yang Y., Wang B., Chang J., Tang Z., Yang B., Lu S. (2021). Insights into Photoluminescence Mechanisms
of Carbon Dots: Advances and Perspectives. Sci.
Bull..

[32] Ehrat F., Bhattacharyya S., Schneider J., Löf A., Wyrwich R., Rogach A. L., Stolarczyk J. K., Urban A. S., Feldmann J. (2017). Tracking the Source
of Carbon Dot
Photoluminescence: Aromatic Domains versus Molecular Fluorophores. Nano Lett..

[33] Arcudi F., D̵ord̵ević L., Prato M. (2019). Design, Synthesis,
and Functionalization Strategies of Tailored Carbon Nanodots. Acc. Chem. Res..

[34] Park Y., Yoo J., Lim B., Kwon W., Rhee S.-W. (2016). Improving the Functionality
of Carbon Nanodots: Doping and Surface Functionalization. J. Mater. Chem. A.

[35] Tepliakov N. V., Kundelev E. V., Khavlyuk P. D., Xiong Y., Leonov M. Yu., Zhu W., Baranov A. V., Fedorov A. V., Rogach A. L., Rukhlenko I. D. (2019). Sp^2^ – Sp^3^ -Hybridized Atomic Domains Determine
Optical Features of Carbon Dots. ACS Nano.

[36] Sciortino L., Sciortino A., Popescu R., Schneider R., Gerthsen D., Agnello S., Cannas M., Messina F. (2018). Tailoring
the Emission Color of Carbon Dots through Nitrogen-Induced Changes
of Their Crystalline Structure. J. Phys. Chem.
C.

[37] Sarkar S., Sudolská M., Dubecký M., Reckmeier C. J., Rogach A. L., Zbořil R., Otyepka M. (2016). Graphitic Nitrogen
Doping in Carbon Dots Causes Red-Shifted Absorption. J. Phys. Chem. C.

[38] Langer M., Paloncýová M., Medved’ M., Otyepka M. (2020). Molecular Fluorophores Self-Organize into C-Dot Seeds
and Incorporate into C-Dot Structures. J. Phys.
Chem. Lett..

[39] Cadranel A., Margraf J. T., Strauss V., Clark T., Guldi D. M. (2019). Carbon
Nanodots for Charge-Transfer Processes. Acc.
Chem. Res..

[40] D’Ambrosio F., Frustaci A., Bodo E. (2025). A Bottom-up
Computational Study of
Doped Amorphous Carbon Clusters as Precursors of Carbon Nanodots. J. Phys. Chem. A.

[41] De
Souza B. (2025). GOAT:A Global Optimization Algorithm for Molecules and Atomic Clusters. Angew. Chem., Int. Ed..

[42] Neese F. (2012). The ORCA Program
System. WIRES Comput. Mol. Sci..

[43] Neese F. (2025). Software Update:
The ORCA Program System—Version 6.0. WIREs Comput. Mol. Sci..

[44] Bannwarth C., Ehlert S., Grimme S. (2019). GFN2-xTB—An Accurate and Broadly
Parametrized Self-Consistent Tight-Binding Quantum Chemical Method
with Multipole Electrostatics and Density-Dependent Dispersion Contributions. J. Chem. Theory Comput..

[45] Bannwarth C., Caldeweyher E., Ehlert S., Hansen A., Pracht P., Seibert J., Spicher S., Grimme S. (2021). Extended Tight-Binding
Quantum Chemistry Methods. WIRES Comput. Mol.
Sci..

[46] Neese F., Olbrich G. (2002). Efficient Use of the Resolution of the Identity Approximation
in Time-Dependent Density Functional Calculations with Hybrid Density
Functionals. Chem. Phys. Lett..

[47] Neese F. (2003). An Improvement
of the Resolution of the Identity Approximation for the Formation
of the Coulomb Matrix. J. Comput. Chem..

[48] Neese F., Wennmohs F., Hansen A., Becker U. (2009). Efficient, Approximate
and Parallel Hartree–Fock and Hybrid DFT Calculations. A ‘Chain-of-Spheres’
Algorithm for the Hartree–Fock Exchange. Chem. Phys..

[49] Helmich-Paris B., De Souza B., Neese F., Izsák R. (2021). An Improved
Chain of Spheres for Exchange Algorithm. J.
Chem. Phys..

[50] Neese F. (2023). The SHARK
Integral Generation and Digestion System. J.
Comput. Chem..

[51] Neese F. (2000). Approximate
Second-Order SCF Convergence for Spin Unrestricted Wavefunctions. Chem. Phys. Lett..

[52] Casanova-Páez M., Goerigk L. (2021). Time-Dependent Long-Range-Corrected
Double-Hybrid Density
Functionals with Spin-Component and Spin-Opposite Scaling: A Comprehensive
Analysis of Singlet–Singlet and Singlet–Triplet Excitation
Energies. J. Chem. Theory Comput..

[53] Garcia-Ratés M., Neese F. (2020). Effect of the Solute
Cavity on the Solvation Energy and Its Derivatives
within the Framework of the Gaussian Charge Scheme. J. Comput. Chem..

[54] Chai J.-D., Head-Gordon M. (2008). Systematic Optimization of Long-Range Corrected Hybrid
Density Functionals. J. Chem. Phys..

[55] Casanova-Páez M., Goerigk L. (2021). Global Double Hybrids Do Not Work for Charge Transfer:
A Comment on “Double Hybrids and Time-dependent Density Functional
Theory: An Implementation and Benchmark on Charge Transfer Excited
States.”. J. Comput. Chem..

[56] Yanai T., Tew D. P., Handy N. C. (2004). A New Hybrid
Exchange–Correlation
Functional Using the Coulomb-Attenuating Method (CAM-B3LYP). Chem. Phys. Lett..

[57] Van
Dijk J., Casanova-Páez M., Goerigk L. (2022). Assessing Recent Time-Dependent
Double-Hybrid Density Functionals on Doublet–Doublet Excitations. ACS Phys. Chem. Au.

[58] Yen T. W., Lai S. K. (2015). Use of Density Functional Theory
Method to Calculate
Structures of Neutral Carbon Clusters *Cn* (3 ≤ *n* ≤ 24) and Study Their Variability of Structural
Forms. J. Chem. Phys..

[59] Ngandjong A. C., Mezei J. Z., Mougenot J., Michau A., Hassouni K., Lombardi G., Seydou M., Maurel F. (2017). Structural Stability
and Growth Mechanism of Neutral and Anionic Small Carbon Clusters:
Density Functional Study. Comput. Theor. Chem..

[60] Dubosq C., Calvo F., Rapacioli M., Dartois E., Pino T., Falvo C., Simon A. (2020). Quantum Modeling
of the Optical Spectra
of Carbon Cluster Structural Families and Relation to the Interstellar
Extinction UV Bump. Astron. Astrophys..

[61] Jia L., Wang Y., Tian X., Wang S., Wang X., Zhang M. (2024). Growth Patterns of Carbon Clusters C _n_ (*n* = 2–60) Identified via ABCluster Searching and DFT Benchmarking. J. Phys. Chem. A.

[62] Shao N., Gao Y., Zeng X. C. (2007). Search for Lowest-Energy
Fullerenes 2: C_38_ to C_80_ and C_112_ to C_120_. J. Phys. Chem. C.

[63] Yutomo E. B., Noor F. A., Winata T. (2021). Effect of
the Number of Nitrogen
Dopants on the Electronic and Magnetic Properties of Graphitic and
Pyridinic N-Doped Graphene – a Density-Functional Study. RSC Adv..

