# Supplementary material for: Investigating the Absorption Properties of Pure and Nitrogen-Doped Carbon Clusters as Models for the Core of Carbon Nanodots
Source: J Phys Chem A. 2026 Feb 4;130(6):1304–16. doi: 10.1021/acs.jpca.5c07923 (PMC12908145; doi:10.1021/acs.jpca.5c07923)
Supplement: Supplementary file 1 [file jp5c07923_si_001.pdf]

# Investigating the Absorption Properties of Pure and Nitrogen-doped Carbon Clusters as Models for the Core of Carbon Nanodots.

*Francesca D'Ambrosio[1], Alice Frustaci[1], Alessandro Azzali[1] Enrico Bodo[1*]*

[1]Chemistry Department, University of Rome "La Sapienza",
P. Aldo Moro 5, 00185, Rome, Italy.

**Supporting Information**

## S1 Assessment of functional choice in TD-DFT spectral calculations

To evaluate the sensitivity of the computed electronic spectra to the choice of the exchange–correlation functional, single-point TD-DFT calculations were carried out for two representative clusters ($C_{19}N$ and $C_{20}$). For $C_{20}$ we compared ωB97X and CAM-B3LYP to ωB2PLYP level, a double hybrid which includes a perturbative treatment of double excitations (CIS(D)) and provides accurate excitation energies for closed- systems. The result is reported in Figure S1.

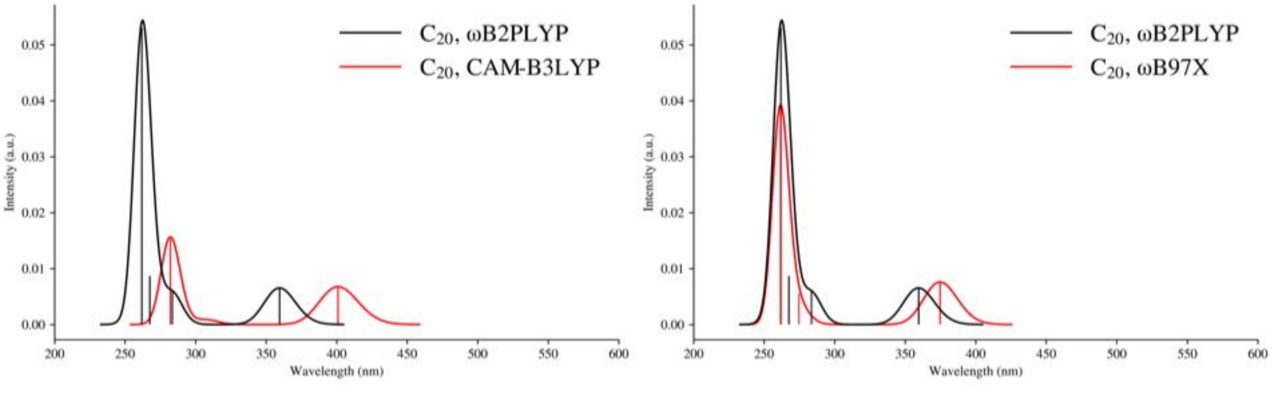


**Figure S1.** TD-DFT calculated spectra on $C_{20}$, using ωB2PLYP as the reference functional. The spectra shape is obtained using a Gaussian broadening of 0.12 eV. Each spectrum is normalized to unit area to allow a direct comparison of relative band positions and intensities.

For $C_{20}$ the range-separated hybrid ωB97X reproduces almost perfectly the reference ωB2PLYP double-hybrid spectra, both in the position ($\Delta E \approx 0.05$–$0.10$ eV) and in the relative oscillator strengths of the main bands, while CAM-B3LYP provides a less intense and blue-shifted spectrum (≈ 0.15–0.20 eV), yet preserving the correct state ordering.

For the open-shell $C_{19}N$ cluster, the doublet–doublet transitions were computed using SOS-ωPBEPP86, which is specifically designed for open-shell excited states. The resulting comparisons of the CAM-B3LYP and ωB97X is reported in Figure S2. For the open-shell $C_{19}N$ cluster, the SOS-ωPBEPP86 functional gives results in excellent agreement with ωB97X, exhibiting the same band pattern and a small red-shift probably due to the spin-scaled CIS(D) correction. The consistent behavior of ωB97X and SOS-ωPBEPP86 indicates that the lowest-lying doublet–doublet excitations are well described by both approaches.

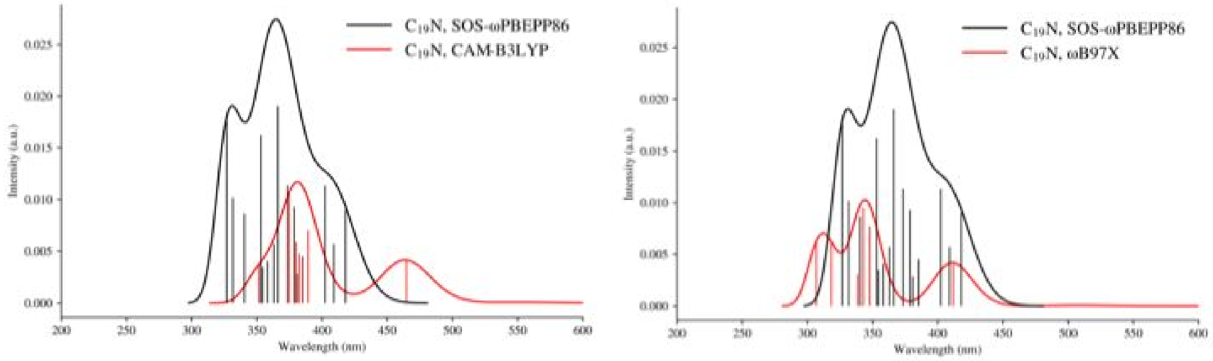


**Figure S2.** TD-DFT calculated spectra on $C_{19}N$, using SOS-ωPBEPP86 as the reference functional. The spectra shape is obtained using a Gaussian broadening of 0.12 eV. Each spectrum is normalized to unit area to allow a direct comparison of relative band positions and intensities.

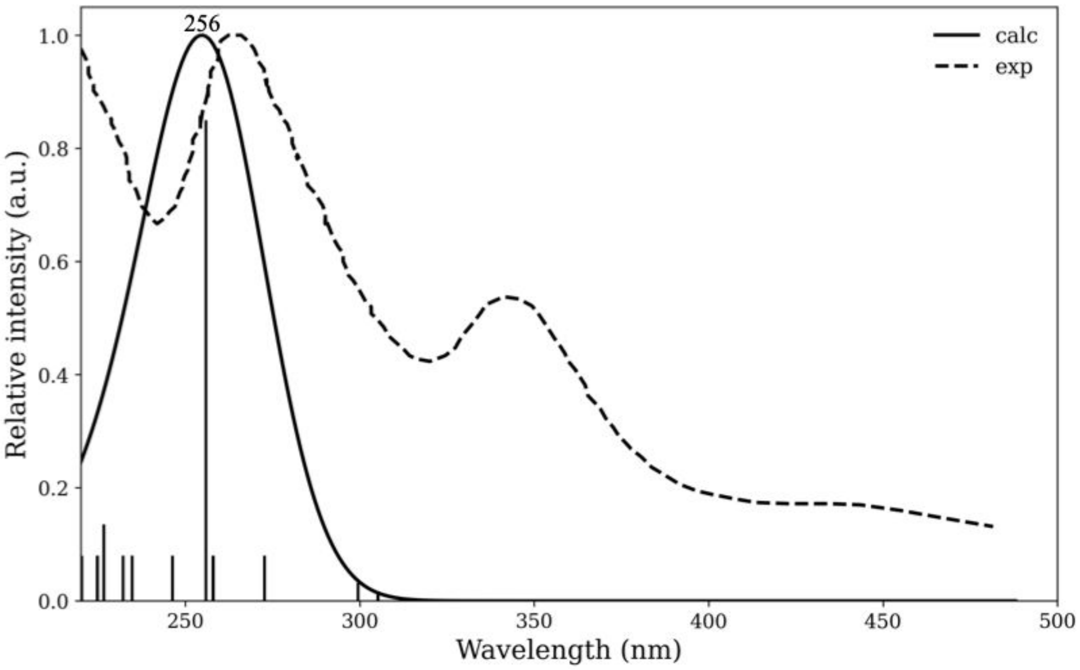


**Figure S3.** UV–vis absorption spectra of $C_{60}$ in water: comparison between experiment and corresponding ωB97X/def2-TZVP calculated spectrum. The experimental data (dashed) was reconstructed from ref. [53] Vertical sticks under the calculated curve mark the positions of the computed electronic transitions. Both spectra are reported in arbitrary units and renormalized to 1. The band at 350 nm is predicted at zero intensity in the TD-DFT spectrum because is activated by vibronic effects due to symmetry breaking.

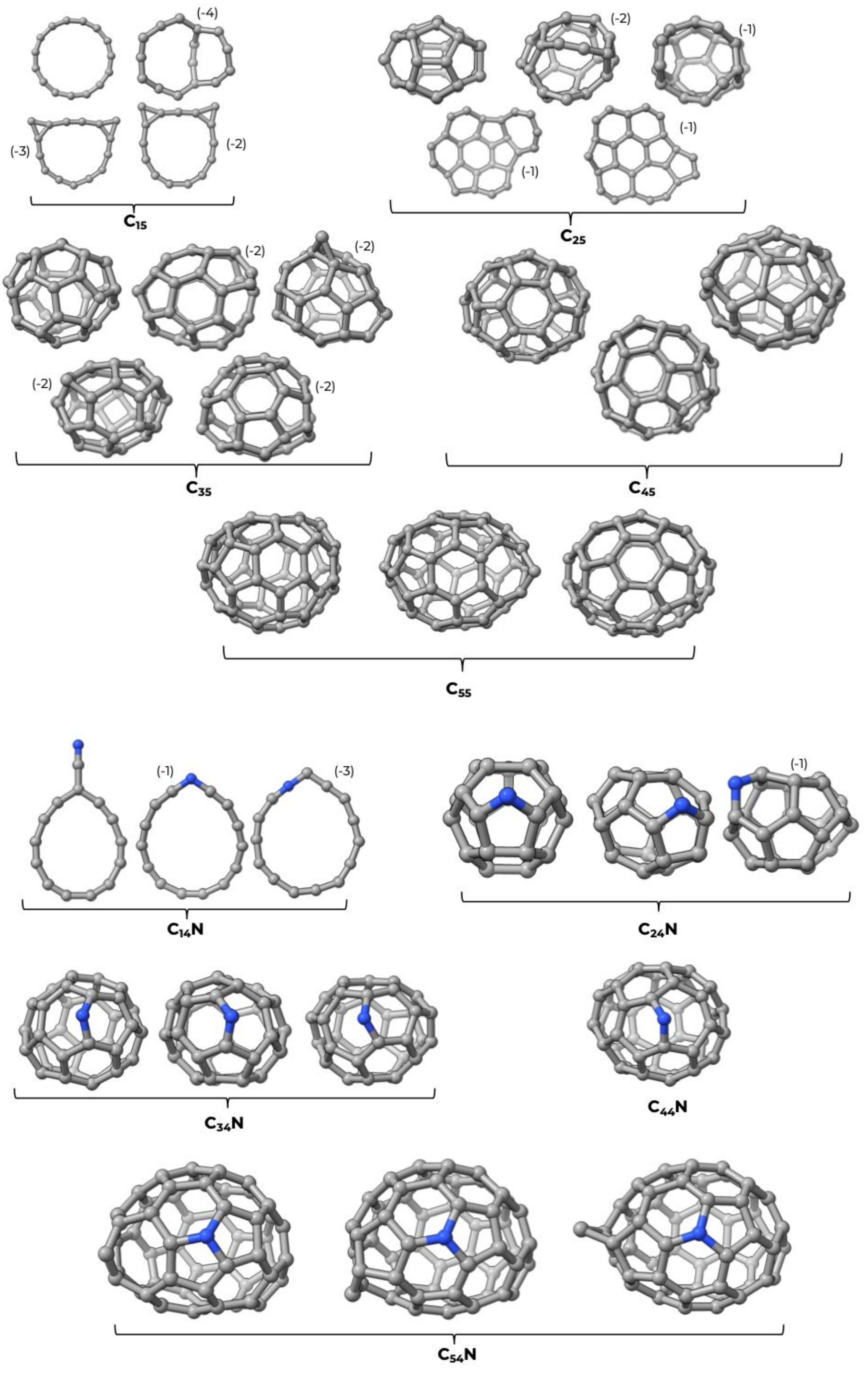


**Figure S4.** Three-dimensional structures for the pure $C_n$ and (top) and doped $C_{n-1}N$ (bottom) clusters. The negative numbers in parentheses indicate the energetic destabilization in terms of atomization energies per atom in kcal/mol with respect to the most stable isomer. Where the difference in atomization energy was less than 1 kcal/mol, we omitted the energy indicator.

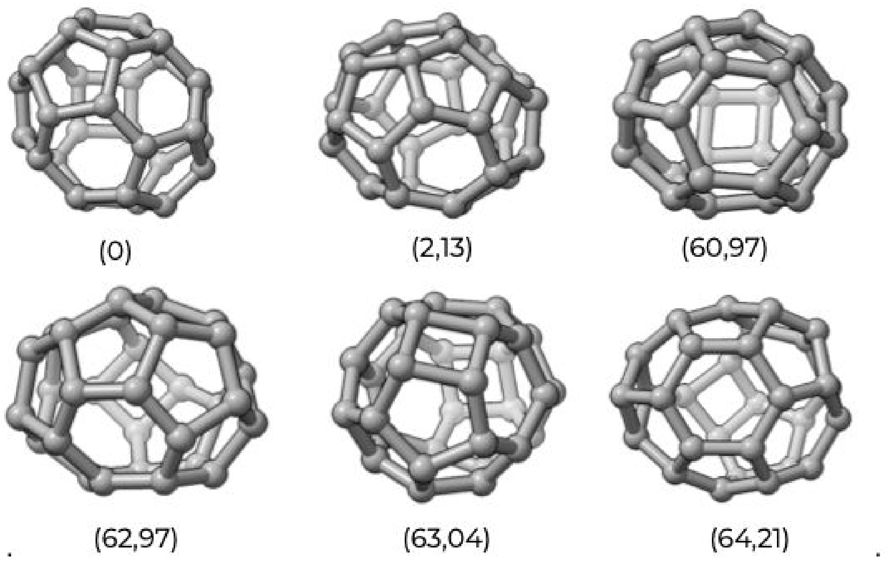


**Figure S5**: The first 6 low-lying isomers of $C_{30}$ obtained from our procedure. The structures have been optimized at the ωB97X-D3BJ/def2-TZVP level and their energy relative to the most stable one is reported in kcal/mol. The second cage from the left at 2.13 kcal/mol over the lowest one, has been created manually using only 6- and 5-memberde rings and optimized. Except this one, all other isomers have a square 4-membered carbon ring. Among these lies the most stable one.

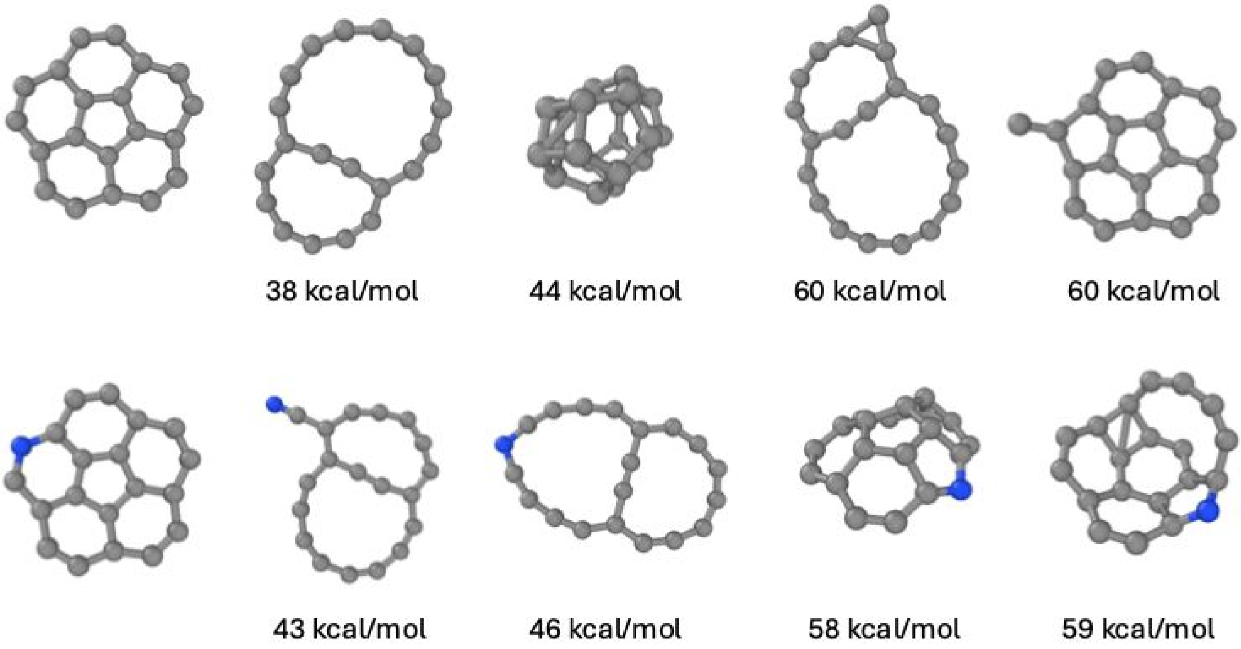


**Figure S6**: Hand-picked high-energy isomers of $C_{20}$ and of $C_{19}N$ obtained from the GOAT procedure at the semiempirical level. Their energy relative to the most stable one is reported in kcal/mol.

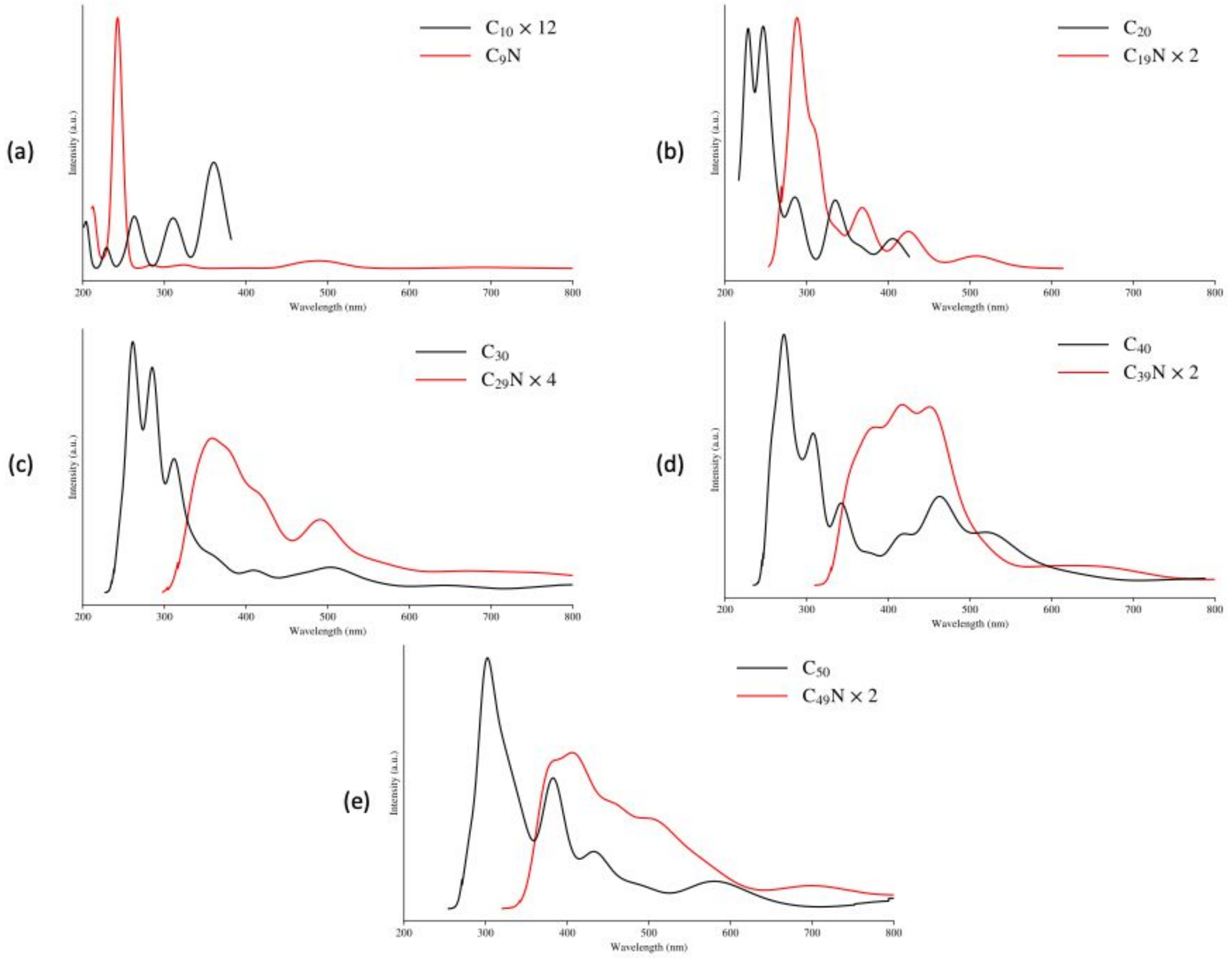


**Figure S7**: TDDFT conformation-averaged spectra at the ωB97X/def2-TZVP level for pure (black lines) and doped (red lines) clusters. These spectra have been obtained by averaging over the spectra of the five lowest energy isomers. The spectra for $C_{10}$ and $C_9N$ have been averaged over three structures due to the lack of other candidates.
